# Coupled dynamics of iron, manganese, and phosphorus in brackish coastal sediments populated by cable bacteria

**DOI:** 10.1002/lno.11776

**Published:** 2021-05-06

**Authors:** Martijn Hermans, Marina Astudillo Pascual, Thilo Behrends, Wytze K. Lenstra, Daniel J. Conley, Caroline P. Slomp

**Affiliations:** ^1^ Department of Earth Sciences (Geochemistry), Faculty of Geosciences Utrecht University Utrecht The Netherlands; ^2^ Department of Geology, Faculty of Science Lund University Lund Sweden; ^3^ Department of Biology and Geology University of Almería Almería Spain

## Abstract

Coastal waters worldwide suffer from increased eutrophication and seasonal bottom water hypoxia. Here, we assess the dynamics of iron (Fe), manganese (Mn), and phosphorus (P) in sediments of the eutrophic, brackish Gulf of Finland populated by cable bacteria. At sites where bottom waters are oxic in spring, surface enrichments of Fe and Mn oxides and high abundances of cable bacteria were observed in sediments upon sampling in early summer. At one site, Fe and P were enriched in a thin layer (~ 3 mm) just below the sediment–water interface. X‐ray absorption near edge structure and micro X‐ray fluorescence analyses indicate that two‐thirds of the P in this layer was associated with poorly crystalline Fe oxides, with an additional contribution of Mn(II) phosphates. The Fe enriched layer was directly overlain by a Mn oxide‐rich surface layer (~ 2 mm). The Fe oxide layer was likely of diagenetic origin, formed through dissolution of Fe monosulfides and carbonates, potentially induced by cable bacteria in the preceding months when bottom waters were oxic. Most of the Mn oxides were likely deposited from the water column as part of a cycle of repeated deposition and remobilization. Further research is required to confirm whether cable bacteria activity in spring indeed promotes the formation of distinct layers enriched in Fe, Mn, and P minerals in Gulf of Finland sediments. The temporal variations in biogeochemical cycling in this seasonally hypoxic coastal system, potentially controlled by cable bacteria activity, have little impact on permanent sedimentary Fe, Mn, and P burial.

Oxygen (O_2_) depletion in bottom waters in coastal areas is expanding worldwide, as a result of excessive nutrient inputs by human activities and climate change (Diaz and Rosenberg 2008; Breitburg et al. 2018). Bottom water hypoxia (O_2_ < 63 *μ*M) and anoxia (O_2_ = 0 *μ*M) have negative consequences for coastal systems. Low bottom water O_2_ can lead to the release of toxic hydrogen sulfide (H_2_S) from sediments into the water column. The lack of O_2_ and the presence of H_2_S in bottom waters can lead to the formation of “dead zones”, which are defined by mass mortality of marine species (Diaz and Rosenberg 2008).

Seasonal hypoxia and anoxia driven by eutrophication greatly impact the biogeochemical cycles of iron (Fe), manganese (Mn), and phosphorus (P) in coastal systems (Ingall and Jahnke 1994; Rabalais et al. 2014; Lenz et al. 2015*a*). When bottom waters are oxic, sedimentary Fe and Mn minerals can form that can sequester dissolved phosphate (HPO_4_^2−^; Conley et al. 2009; Turnewitsch and Pohl 2010). During periods of hypoxia/anoxia, these Fe and Mn minerals which mostly consist of oxides or (oxy)hydroxides (collectively referred to as oxides) can reductively dissolve, leading to release of dissolved Fe, Mn, and HPO_4_^2−^ from sediments to the overlying water (Burdige 1993; Conley et al. 2002). The recycling of HPO_4_^2−^ from the sediment can contribute to a high primary productivity in surface waters and thereby to high rates of organic matter supply to the sediment. This ultimately may sustain bottom water hypoxia even when riverine inputs of P are reduced (Conley et al. 2002).

Recently, a novel type of multicellular filamentous bacteria, belonging to the *Desulfobulbaceae* family was discovered (Nielsen et al. 2010; Pfeffer et al. 2012). These so‐called “cable bacteria” can strongly enhance the formation of Fe and Mn oxides in surface sediments (Risgaard‐Petersen et al. 2012; Seitaj et al. 2015; Sulu‐Gambari et al. 2016*a*), and thereby impact the sedimentary P cycle (Sulu‐Gambari et al. 2016*b*). Cable bacteria couple the oxidation of dissolved H_2_S in deeper sediment layers to the reduction of O_2_ or nitrate (NO_3_^−^) near the sediment surface by transporting electrons along their filaments over cm‐scale distances (Pfeffer et al. 2012; Marzocchi et al. 2014). Their metabolic activity leads to the formation of a suboxic zone (i.e., where O_2_ and H_2_S are both absent) and a unique pH profile (Nielsen et al. 2010), defined by a pH increase (~ 9) near the sediment–water interface and a relatively low pH (< 6.5) in the suboxic zone (Pfeffer et al. 2012). The strong acidification leads to dissolution of iron monosulfide (FeS; Risgaard‐Petersen et al. 2012; Seitaj et al. 2015) and Fe, Mn, and calcium (Ca) carbonates (Sulu‐Gambari et al. 2016*a*). When the dissolved Fe and Mn released from mineral dissolution diffuse upward, Fe and Mn oxides can form upon contact with O_2_ (Risgaard‐Petersen et al. 2012; Sulu‐Gambari et al. 2016*a*), or, for dissolved Fe, upon contact with Mn oxides (Wang and Van Cappellen 1996; Sulu‐Gambari et al. 2016*a*,*b*). Hence, the metabolic activity of cable bacteria can lead to a pronounced redistribution of reactive Fe and Mn in aquatic sediments. Vertical redistributions of Fe and Mn in sediments are not exclusively tied to cable bacteria activity. However, the enhanced dissolution of Fe and Mn minerals due to H^+^ production by cable bacteria, upon their establishment following an environmental perturbation, typically leads to a relatively stronger focusing of Fe and Mn oxides in the (sub‐)surface sediment, when compared to sediments where cable bacteria are absent (Risgaard‐Petersen et al. 2012; Hermans et al. 2020).

The increased formation of Fe and Mn oxides induced by cable bacteria can have major biogeochemical impacts at the system scale, as shown in a study for a seasonally hypoxic marine basin, Lake Grevelingen, where the metal oxides formed through their metabolic activity in spring buffered the release of H_2_S and HPO_4_^2−^ from the sediment during hypoxia in summer (Seitaj et al. 2015; Sulu‐Gambari et al. 2016*b*). Recently, the same buffer mechanism for H_2_S was suggested to be active in sediments of the eutrophic, brackish Gulf of Finland, in an area subject to seasonal hypoxia (Hermans et al. 2019). Here, the activity of cable bacteria may explain why bottom waters seldom become anoxic and sulfidic (“euxinic”) during peak hypoxia in summer (Hermans et al. 2019). Besides their impact on sedimentary Fe and Mn cycling, cable bacteria may also affect water column dynamics of Fe and Mn by promoting repeated cycles of mobilization of Fe and Mn in dissolved form in the sediment pore water, release to the overlying water and oxidation upon contact with O_2_ followed by deposition of Fe and Mn oxides, which is referred to as “refluxing” (Adelson et al. 2001; Sulu‐Gambari et al. 2017). The impact of cable bacteria on the dynamics of P in the Gulf of Finland is not known, although an effect is expected (Sulu‐Gambari et al. 2016*b*).

Here, we assess the coupled dynamics of Fe, Mn, and P in the water column and sediment at three sites in the Gulf of Finland with contrasting bottom water redox conditions. Using a combination of geochemical pore water and sediment analyses, including X‐ray spectroscopy, we address whether and how sedimentary Fe and Mn minerals at these sites contribute to P sequestration and specifically the potential role of cable bacteria therein. Water column data and in‐situ measured benthic fluxes of Fe, Mn, and P are used to assess the potential impact of cable bacteria on the water column chemistry at our study sites. Given their high abundance in these sediments, we suggest that cable bacteria likely affect Fe, Mn, and P dynamics near the sediment–water interface in the Gulf of Finland, but permanent burial of these elements is not affected.

## Methods

### Study area

The Baltic Sea has received excessive riverine inputs of P and nitrogen (N) over the last century resulting in enhanced primary production and an increase in the spatial extent of bottom water O_2_ depletion (Gustafsson et al. 2012). The Baltic Sea is now considered the world's largest human‐induced hypoxic/anoxic water body (Carstensen et al. 2014).

**Fig. 1:**
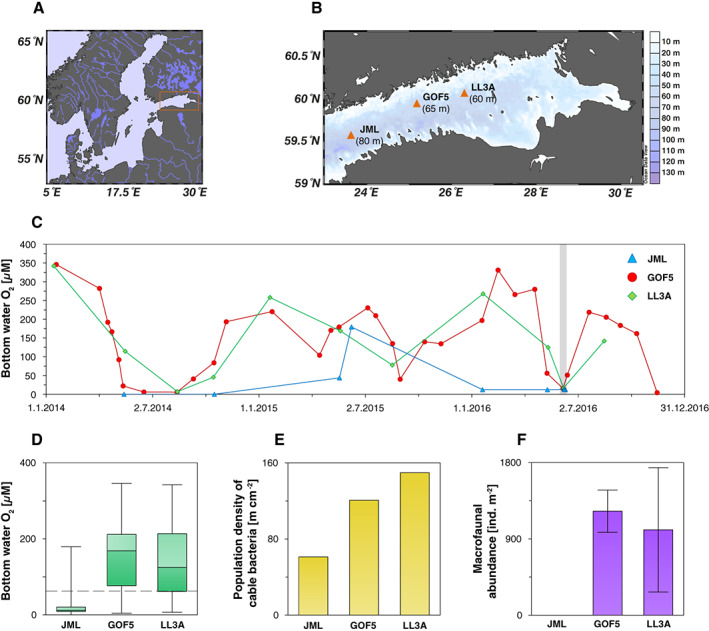
Study area: (**A**) map of the Baltic Sea, the orange rectangle highlights our study area. (**B**) Bathymetric map of the Gulf of Finland indicating the locations of the three stations. Water depths: JML: 80 m, GOF5: 65 m, LL3A: 60 m. (**C**) Bottom water O_2_ concentrations for 2014–2016 derived from the HELCOM database. The gray bar indicates the time interval when our sampling campaign took place. The data points within this gray bar were retrieved during our expedition. (**D**) Range in bottom water O_2_ for 2014–2016 derived from the HELCOM database. The black dashed line represents the hypoxic boundary (63 *μ*M). The solid line between the boxes represents the median, whereas the boxes are the lower and upper quartile, respectively. The error bars indicate the minimum and maximum O_2_ levels. (**E**) Population density of cable bacteria, meters of cable bacteria filament per square centimeter, [m cm^−2^] integrated over the top 2.5 cm of the surface sediment collected in June 2016 (Hermans et al. 2019). (**F**) Macrofaunal abundance [ind. m^−2^] in June 2016 integrated over a sediment depth of ~ 12 cm (Hermans et al. 2019). The error bars indicate the standard deviation. Macrofauna were not detectable at site JML.

The Gulf of Finland is the most eutrophic basin of the Baltic Sea (Fig. 1A; HELCOM 2009; Carstensen et al. 2014). The absence of a sill between the Baltic Proper and Gulf of Finland allows exchange of large water volumes between the two basins resulting in a maximum residence time of water in the Gulf of Finland of ~ 2 yr (Andrejev et al. 2004). Strong westerly and south‐westerly winds push surface water from the main basin into coastal areas of the Gulf of Finland, with a compensating outflow of deeper water. As a consequence, stratification frequently decreases or completely collapses during winter (Elken et al. 2014).

Our three study sites (JML, GOF5, and LL3A; salinities 9–11) are located along a water depth gradient ranging from 60 to 85 m water depth in the Gulf of Finland (Fig. 1B; Table 1) and differ in terms of bottom water O_2_ conditions. From 2014 to 2016, site JML was mostly hypoxic (O_2_ < 63 *μ*M) and only briefly became oxic (O_2_ > 63 *μ*M) in June 2015 (Fig. 1C,D; Hermans et al. 2019). In contrast, sites GOF5 and LL3A were characterized by a seasonal cycle with high bottom water O_2_ most of the year and low bottom water O_2_ in summer (Fig. 1C,D; Hermans et al. 2019). The sediments were populated by cable bacteria at sites GOF5 and LL3A in June 2016 (Fig. 1E; Hermans et al. 2019). Site JML had a much lower abundance of cable bacteria (61 m cm^−2^) than GOF5 and LL3A (121 and 150 m cm^−2^, respectively) as a consequence of the lower O_2_ availability (Hermans et al. 2019). The abundances at sites GOF5 and LL3A were much higher than elsewhere in the Baltic Sea (Hermans et al. 2019), and comparable to abundances found at field sites with active cable bacteria (Malkin et al. 2017). High‐resolution pore water profiles of pH, O_2_, and ΣH_2_S (H_2_S + HS^−^ + S^2−^) at these sites did not indicate activity of cable bacteria at the time of sampling, as a consequence of the recent transition from oxic to hypoxic/anoxic bottom waters, and the corresponding low O_2_ concentrations observed near the sediment–water interface (Hermans et al. 2019). Sites GOF5 and LL3A were both characterized by a suboxic zone of ~ 7 mm (Hermans et al. 2019). At site GOF5, the activity of cable bacteria could be reactivated within 5 d upon bottom water reoxygenation of an intact sediment core (Hermans et al. 2019). Macrofauna (< 0.5 mm) were absent at site JML, whereas relatively low macrofaunal abundances (1227 and 1007 ind. m^2^) were observed at sites GOF5 and LL3A. At these latter sites, the polychaete *Marenzelleria* dominated (Fig. 1F; Hermans et al. 2019). Despite the low macrofaunal abundances at these sites, significant activity of meiofauna may be present (Broman et al. 2020).

**Table 1:** Site characteristics in June 2016.

Site	Water depth (m)	Coordinates (N, E)	Sedimentation rate (cm yr^−1^)	Total P burial rate (mmol m^−2^ yr^−1^)	Org. P burial rate (mmol m^−2^ yr^−1^)	Salinity	Bottom water O_2_ (*μ*M)	Areal density of cable bacteria (m cm^−3^)^*^
JML	80	59°34.92′ 23°37.50′	0.4	43	12	10.7	0	61
GOF5	65	59°57.10′ 25°11.02	0.7	89	29	9.4	10	121
LL3A	60	60°4.43′ 26°18.30′	1.5	147	55	8.9	5	150

* Data from Hermans et al. (2019).

### Water column sampling and analyses

In June 2016, sites JML, GOF5, and LL3A were sampled during a research expedition with R/V *Pelagia*. Water column depth profiles of temperature, salinity and density were obtained with an ultraclean CTD‐system. Dissolved O_2_ was measured with a Sea‐Bird O_2_ sensor attached to a CTD. Water column samples were collected using 24 ultratrace metal clean PVDF samplers of 24 liters each, placed in two rows of 12 samplers mounted onto a rectangular titanium frame (De Baar et al. 2008; Rijkenberg et al. 2015). The CTD system was deployed with a Kevlar hydrowire and nitrogen pressure was applied at the top of the sampler during sample collection. At the time of sampling, the water column was stratified at all sites, as evident from the temperature, salinity and density profiles (Fig. S1).

Samples for dissolved HPO_4_^2−^, H_2_S, and NO_x_ (NO_3_ + NO_2_) were obtained using 20 mL syringes and passed through 0.45 *μ*m nylon filters. Aliquots for H_2_S analysis were transferred into N_2_ purged 8 mM NaOH solutions immediately after filtration to prevent loss of H_2_S. Subsequently, the HPO_4_^2−^ and H_2_S concentrations were determined via the methylene and molybdate blue complex methods, respectively (Grasshoff et al. 2009), using QuAAtro (Bran + Luebbe) gas‐segmented continuous flow analyzers on‐board ship. Concentrations of NO_x_ were measured using imidazol buffer following Grasshoff et al. (2009).

Acid‐washed LDPE bottles (Nalgene™) were rinsed three times with sample prior to sample collection. Unfiltered samples for total dissolvable Fe, Mn, and aluminum (Al) and filtered samples for dissolved Fe, Mn, and Al (0.2 *μ*m Sartobran 300 cartridge, Sartorius) were acidified as quickly as possible after sampling to pH 1.8 using distilled HCl and stored at 4°C until analysis (within 1 yr). This acidification can potentially lead to an underestimation of total dissolvable Fe and Mn as a consequence of precipitation of humic substances upon acidification (Oldham et al. 2017). Total dissolvable Fe concentrations were determined by flow injection using a mixture of luminol and triethylenetetramine with preconcentration on an iminodiacetic acid resin (Rijkenberg et al. 2014). The blank was 25 ± 1 pM (*n* = 30). To ensure high accuracy of the measurements, the system was checked daily using a GEOTRACES standard or an in‐house reference material (acidified sea water pH 1.8). For GEOTRACES SAFe D1, we obtained measurements with an average value of 0.69 ± 0.26 nM (consistent with the community consensus value of 0.67 ± 0.04; Johnson et al. 2007). Total dissolvable Mn and total Al concentrations were determined by Inductively Coupled Plasma‐Mass Spectrometry (ICP‐MS; Nexion Perkin Elmer), after online sample pretreatment using a SC‐DX SeaFAST S2 (Elemental Scientific) as described in Lagerström et al. (2013). The blank for Mn was 1.012 ± 0.155 nM, three reference materials were used (NASS‐6, SLRS and SLEW, and the overall recovery rate was 102.4 ± 10.6%, *n* = 24). The detection limit for Al was 4.3 ± 3.4 nM (*n* = 5) and the value of the blank was 1.98 ± 0.41 nM (*n* = 84).

At sites GOF5 and LL3A, suspended matter samples from the water column were retrieved from the PVDF samplers using 0.2 *μ*m Supor membrane filters which were connected to the samplers at 60 and 58 m water depth, respectively. Approximately, 5 liters of sample was passed through the filters for suspended matter collection.

Monthly time‐series data for bottom water O_2_ and HPO_4_^2−^ for 2002–2017 were obtained from the Swedish Ocean Archive (SHARK) database at the Swedish Meteorological and Hydrological Institute (SMHI; http://sharkweb.smhi.se).

### Sediment and pore water collection

Sediment cores (diameter 10 cm) were collected in a single cast using a multi corer (Oktopus GmbH, Germany). Duplicate bottom water samples were retrieved directly from the overlying water. At each site, one sediment core was sectioned immediately after core retrieval in a glovebox under a N_2_ atmosphere (0.5–4 cm resolution) in a climate‐controlled laboratory on‐board ship at in situ temperature. Slices for each depth interval were split into vials for porosity and solid‐phase analysis and polypropylene 50 mL tubes that were centrifuged at 3500 rpm for 20 min at in situ temperature for pore water retrieval. All sample handling took place in an inert atmosphere, hence oxidation artifacts can be excluded (Kraal et al. 2009). The sediment was stored under a N_2_ atmosphere at − 20°C. Additional cores were used for high‐resolution depth profiling of pore water pH, O_2_, and ΣH_2_S, sediment micro X‐Ray Fluorescence (*μ*XRF) and X‐Ray Absorption Spectroscopy (XAS; sites JML and GOF5 only) and the determination of sedimentation rates using ^210^Pb dating.

Bottom water samples and the supernatant from the centrifuge tubes (~ 5 mL) were passed through 0.45 *μ*m nylon filters under anoxic conditions. Directly after filtration, subsamples of 0.5 mL for H_2_S analysis were trapped in an 8 mM NaOH solution (1.5 mL). Concentrations of H_2_S and HPO_4_^2−^ were determined as described for the water column samples. Ammonium (NH_4_^+^) concentrations were determined on‐board ship using a QuAAtro (Bran + Luebbe) gas segmented continuous flow analyzer using the phenol‐hypochlorite method (Koroleff 1969). Samples for metals were acidified on‐board with 10 *μ*L Suprapur^®^ HCl (35%) per mL sample and analyzed for dissolved Fe and Mn onshore using Inductively Coupled Plasma‐Optical Emission Spectroscopy (ICP‐OES, Spectro Arcos). Sulfate (SO_4_^2−^) and chloride (Cl^−^) concentrations were determined using ion chromatography (IC).

### Benthic and diffusive fluxes

Fluxes of dissolved NH_4_^+^, HPO_4_^2−^, Fe, and Mn across the sediment–water interface were determined in situ, using two benthic landers, equipped with three chambers, each with a square surface area of 144 cm^2^ and a volume of overlying water ranging from 0.9 to 2 liters. During incubation, the overlying water was stirred continuously as described in Lenstra et al. (2019). Fluxes of dissolved NH_4_^+^, HPO_4_^2−^, Fe, and Mn derived from the lander's incubation chambers were averaged. Particularly for Fe, directly measured fluxes are frequently highly variable and should be interpreted with caution (Aller 1980).

Diffusive fluxes of dissolved NH_4_^+^, HPO_4_^2−^, Fe, and Mn across the sediment–water interface were determined using Fick's first law (Supplementary Information 1.2) as described in Berner (1980). In this calculation, dissolved Fe and Mn were assumed to be Fe^2+^ and Mn^2+^, although Mn^3+^ (Madison et al. 2013) or colloidal and nanoparticulate Fe and Mn might also have been present (Raiswell and Canfield 2012; Oldham et al. 2017). Note, however, that organic complexes and ion pairs were not considered, and no explicit corrections for potential electric fields associated with the activity of cable bacteria were made.

### Sediment analyses

Sediments were freeze‐dried, ground, and stored under anoxic conditions to prevent oxidation artifacts (Kraal et al. 2009). Porosity was determined from the weight loss upon freeze‐drying, using a sediment density of 2.65 g cm^−3^ (Burdige 2006). Salt corrections were applied to all solid‐phase data, using the gravimetric water content and salinity to determine the amount of salt after freeze‐drying.

For the analysis of total Fe, Mn, S, and P contents, aliquots of ~ 125 mg freeze‐dried sediment were digested in a mixture of HF (40%) and 2.5 mL HClO_4_‐HNO_3_ (ratio 3 : 2) in closed PTFE vessels. The residual gel was dissolved in 1 M HNO_3_ and the Fe, Mn, S, and P contents were determined by ICP‐OES.

Aliquots of ~ 300 mg of freeze‐dried sediment were decalcified using two wash‐steps of 1 M HCl for 24 h (van Santvoort et al. 2002). After drying and re‐powdering, the decalcified samples were analyzed for their carbon and nitrogen contents using a Fisons Instruments NA 1500 NCS analyzer. Organic carbon (C_org_) and nitrogen (N_org_) contents were corrected for the weight loss during decalcification.

Iron phases were fractionated using aliquots of ~ 80 mg freeze‐dried sediment. A combination of two operational sequential extraction methods after Poulton and Canfield (2005) and Claff et al. (2010) was applied to fractionate the Fe phases into: [I] labile ferrous Fe (siderite [FeCO_3_], FeS and ferrous clays such as glauconite) and ferric Fe minerals using 1 M HCl for 4 h, [II] crystalline Fe minerals using citrate dithionite buffer (CDB) for 4 h, [III] magnetite (Fe_3_O_4_) using 0.2 M (NH_4_)_2_C_2_O_4_/0.17 M C_2_H_2_O_4_ for 6 h, and [IV] pyrite (FeS_2_) extracted by concentrated HNO_3_ for 2 h. The sum of these four fractions is defined as total extractable Fe.

For sedimentary P speciation, the sequential extraction procedure after Ruttenberg (1992) as modified by Slomp et al. (1996) was applied using aliquots of ~ 150 mg freeze‐dried sediment. Sediment P was fractionated into: [I] exchangeable‐P (1 M MgCl_2_; 0.5 h), [II] CDB‐P (citrate‐bicarbonate‐CBD buffered with a mixture of Na citrate and Na bicarbonate to pH 7.5; 8 h), [III] authigenic‐P associated with calcium carbonate (CaCO_3_) + carbonate fluorapatite + biogenic hydroxyapatite (1 M Na‐acetate buffered with acetic acid to pH 4, 6 h), [IV] detrital‐P (1 M HCl, 24 h), and [V] organic‐P (ashed at 550°C for 2 h following extraction with 1 M HCl for 24 h).

Sulfide was fractionated using aliquots of ~ 300 mg freeze‐dried sediment following the sequential, passive distillation extraction procedure after Burton et al. (2006, 2008) as modified by Kraal et al. (2013). [I] FeS (Acid Volatile Sulfur (AVS, representing FeS; 6 M HCl; 24 h, [II] elemental sulfur (S_0_; methanol; 16 h), and [III] chromium reducible sulfur (CRS, representing FeS_2_; 500 g L^−1^ chromium(II) chloride in 32% HCl; 48 h).

Values of several environmental indicators were calculated from the sediment geochemical analyses: (1) S/C_org_, as an indicator of bottom water salinity (Berner and Raiswell 1984), (2) degree of sulfidization (DOS) and pyritization (DOP), (3) Fe/Al and (4) the ratio of highly reactive Fe and total Fe (FeHR/total Fe; Raiswell et al. 2018), which are all proxies for bottom water redox conditions and (5) Py‐Fe(II) and (6) Sid‐Fe(II), which indicate the proportion of Fe(II) that is bound as pyrite and siderite, respectively (Aller et al. 2004).

### Sediment accumulation and P burial rates

Freeze‐dried sediment samples were analyzed for ^210^Pb by direct gamma counting at 46.5 keV with a high purity germanium detector (Ortec GEM‐FX8530P4‐RB). Self‐absorption was measured directly and the detector efficiency was verified with a National Institute of Standards and Technology standard. Excess ^210^Pb was derived from the difference between the measured total ^210^Pb and the estimate of the supported ^210^Pb activity as given by ^214^Pb (^210^Pb_exc_ = ^210^Pb_total_ − ^214^Pb). Sedimentation rates were estimated by fitting a reaction transport model (Soetaert and Herman 2008) to the ^210^Pb depth profiles taking the depth dependent changes in porosity into account. The reaction transport model assumes steady state. Hence, the calculated sedimentation rates are approximations. Total Fe, Mn, and P burial was calculated as a function of the sedimentation rate, total Fe, Mn and P at depth in the sediment and the porosity (Supplementary Information 1.3).

### Epoxy embedding and *μ*XRF mapping

At sites JML and GOF5, subcores (first 7 cm of surface sediment retrieved as a small cylinder from an intact sediment multicore) were embedded with epoxy resin for high‐resolution elemental mapping (Jilbert and Slomp 2013). After curing the epoxy‐embedded cores were split vertically using a rock saw. The embedded core was polished by applying a 0.3 *μ*m layer of alumina powder. High‐resolution elemental maps of Fe, Mn, P, Ca, and S (30 *μ*m spot size) were obtained using a Desktop EDAX Orbis *μ*XRF analyzer (Rh tube set at 30 kV, 500 *μ*A, 300 ms dwell‐time, equipped with a poly‐capillary lens).

### Synchrotron‐based XAS and *μ*XRF mapping

Suspended matter samples from the water column (Supplementary Information 1.4) and epoxy embedded sediment retrieved from site GOF5 were further investigated for their Fe and Mn mineralogy at the ID21 beamline (Salomé et al. 2013) at the European Synchrotron Radiation Facility (ESRF) in Grenoble, France, combining X‐ray fluorescence and X‐ray Absorption Spectroscopy (XAS). High‐resolution *μ*XRF maps of Fe and Mn were processed using the PyMca X‐ray Fluorescence Toolkit (Solé et al. 2007). Calibration of the monochromator energy was performed using the maximum intensity of the first derivative of Fe foil at 7.11198 keV for Fe and Mn foil at 6.53862 keV for Mn. The size of the X‐ray beam (0.35 × 0.80 *μ*m) was controlled by a Kirkpatrick–Baez mirrors system. Although the cross section of the beam was very narrow, the analyzed volume in the sediment sample was relatively large. To illustrate this, the absorption length, calculated with the program Hephaestus (Ravel and Newville 2005) of a 7 keV beam in silica (SiO_2_) with a density of 2.2 g cm^−3^ is ~ 85 *μ*m. This implies that Fe and Mn atoms, which are located below a depth of several tenths of micrometers below the sample's surface, can still contribute to the measured fluorescence spectrum, although the signal's intensity decreases with depth.

Hotspots of Fe and Mn selected from the synchrotron‐based maps were further subjected to XAS analysis to determine the Fe and Mn mineralogy at these spots/areas. Spectra to investigate the X‐ray Absorption Near Edge Structure (XANES) were collected within the energy range, 7.00–7.65 keV for Fe and 6.50–6.90 keV for Mn. The XANES spectra were corrected for the background signal and normalized using the ATHENA software package (Ravel and Newville 2005). The ATHENA software package was also used for Linear Combination Fitting (LCF) of the spectra. The normalized Fe and Mn spectra were subjected to Iterative Transformation Factor Analysis (ITFA) using the ITFA software package as described in Rossberg et al. (2003) for principal component analysis of the spectra.

The Fe content in the suspended matter samples was too low to obtain XAS data of sufficient quality, therefore only those of Mn are presented. XAS spectra were collected with a continuously moving monochromator, implying that acquisition time and energy resolution were not adjusted depending on energy range. The latter is typically used to improve the signal to noise ratio for analyzing the Extended X‐ray Absorption Fine Structure (EXAFS) of spectra. For this reason, the quality of Mn spectra was insufficient for EXAFS analysis and the energy range of Fe EXAFS spectra was limited. Although limited, when available, Fe EXAFS spectra for epoxy embedded sediments were used to better constrain the LCF.

## Results

### Water column geochemistry

**Fig. 2:**
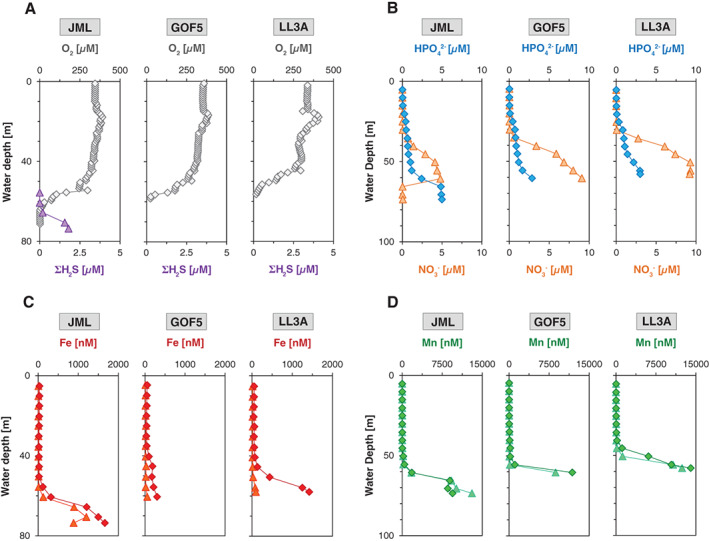
Water column depth profiles of (**A**) dissolved O_2_ (gray) and ΣH_2_S (purple), (**B**) dissolved HPO_4_^2−^ (blue) and NO_3_^−^ (orange), (**C**) total dissolvable (*d*) Fe (red; diamonds) and dissolved Fe (orange; triangles), and (**D**) total dissolvable (*d*) Mn (green; diamonds) and dissolved Mn (cyan; triangles) at sites JML, GOF5, and LL3A in June 2016.

At all sites, O_2_ was near‐saturation in the upper ~ 50 m of the water column. Below 50 m, O_2_ decreased strongly from ~ 350 to ~ 0 *μ*M with increasing water depth. Sulfide was only observed in the lower part of the water column and bottom water at site JML, reaching concentrations up to 18 *μ*M (Fig. 2A). Nitrate concentrations were < 0.1 *μ*M in the upper ~ 30 m of the water column and increased with water depth to values of ~ 5 (JML) or ~ 9 *μ*M (GOF5 and LL3A) until ~ 60 m. At site JML, NO_3_ concentrations decreased to values < 0.1 *μ*M. At all sites, HPO_4_^2−^ increased with water depth from 0.02 up to ~ 5 *μ*M (Fig. 2B). Total dissolved and dissolvable Fe and Mn concentrations were relatively low in the upper ~ 50 m of the water column, and highest near the seafloor, with the latter reaching values up to ~ 1700 and ~ 14.000 nM, respectively (Fig. 2C,D). Concentrations of total dissolvable Fe were generally 4‐ to 37‐fold lower compared to those of Mn. At sites GOF5 and LL3A, the difference between total dissolvable and dissolved Fe and Mn can be used as a measure of particulate Fe and Mn. This is not possible for samples below the redoxcline at site JML, since nanoparticulate FeS can pass through a 0.2 *μ*m filter (Lenstra et al. 2021). Our results for sites GOF5 and LL3A imply that most Fe is present in particulate form, whereas a large proportion of the Mn is present in dissolved form. Particulate Fe and Mn concentrations were 10‐fold lower than and equal to dissolved Mn at sites GOF5 and LL3A, respectively (Fig. 2C,D). At site GOF5, particulate Fe/Al and Mn/Al ratios (wt%/wt%) close to the seafloor were equal to 20 and 222, respectively. At site LL3A, we observed Fe/Al and Mn/Al ratios of 165 and 167, respectively (Fig. S2).

**Fig. 3:**
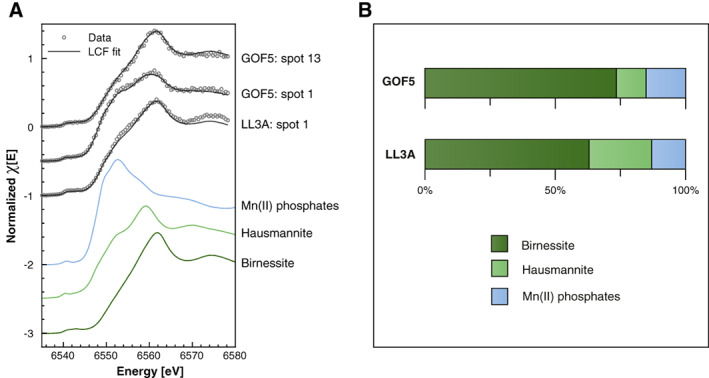
Synchrotron‐based Mn analysis for suspended matter in the water column at sites GOF5 and LL3A. (**A**) Mn XANES spectra from three spots selected from the water column filters (Fig. S3). The selected spectra represent the most diverting spectra based on component analysis. The gray circles represent the actual XANES measurements, whereas the black line was obtained through linear combination fitting using the spectra of the three reference materials: birnessite (Mn(IV)), hausmannite (Mn(II/III) and Mn(II)) phosphates. (**B**) Relative proportion of Mn phases in the suspended matter.

For satisfactory reproduction of the Mn XANES spectra for the suspended matter samples from the water column at sites GOF5 and LL3A, the combination of at least three reference spectra was required. When using the combinatorics option in LCF using ATHENA, the best result was obtained when using the Mn oxides, birnessite and hausmannite, and a Mn(II) phosphate standard (hureaulite; Manceau et al. 2012; Fig. 3A; Fig. S3). The position of the absorption maximum of our spectra was close to that of Mn in birnessite and hence indicates the predominant presence of Mn(IV). However, the spectra differ with respect to the shape and position of the edge, which is shifted to lower energies when compared to birnessite, which points toward the presence of Mn with a lower oxidation state. Based on LCF, we estimated that Mn in suspended matter was predominantly present in the form of Mn(IV) (57–67%), with Mn(III) and Mn(II) contributing the remainder of the Mn (33–43%; Fig. 3B). The results from LCF further suggested that the Mn(IV) occurs in the form of phyllo‐ or tectomanganates with predominantly edge sharing Mn octahedra, such as birnessite or todorokite (and not pyrolusite). This implies that most Mn in the suspended matter was present in the form of Mn(IV) accompanied with Mn(II) and possibly some minor amounts of Mn(III).

### Pore water profiles

**Fig. 4:**
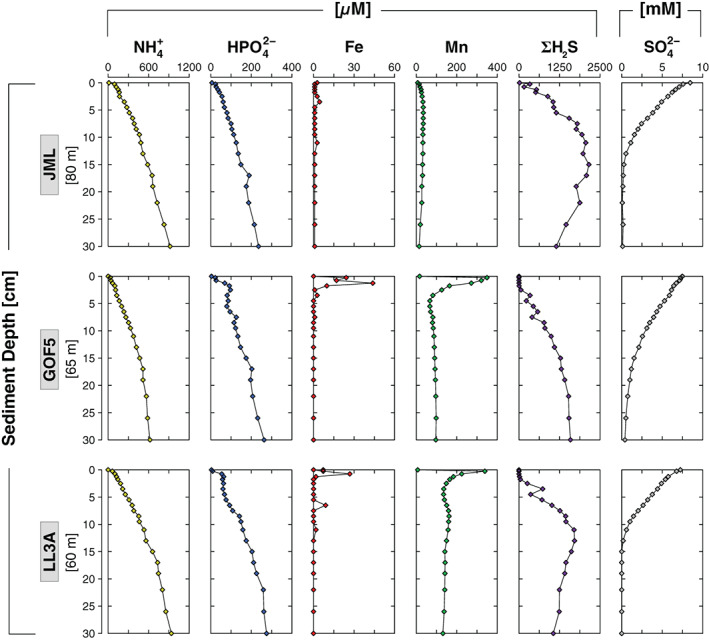
Porewater profiles of dissolved NH_4_^+^, HPO_4_^2−^, Fe, Mn, ΣH_2_S and SO_4_^2−^ for the upper 30 cm of the sediment at sites JML, GOF5, and LL3A in June 2016.

At all sites, NH_4_^+^ and HPO_4_^2−^ in the pore water increased with depth reaching concentrations of up to ~ 900 and ~ 250 *μ*M, respectively (Fig. 4). Phosphate was removed within the top ~ 1 cm of the sediment at sites GOF5 and LL3A. Dissolved Fe and Mn were low at site JML, whereas sites GOF5 and LL3A were characterized by subsurface peaks of dissolved Fe and Mn with maximum concentrations of up to ~ 44 and ~ 350 *μ*M, respectively. Sulfide increased within the first 15 cm of the sediment at all sites with maximum concentrations of up to ~ 2100 *μ*M. Sulfide decreased again below 15 cm depth at sites JML and LL3A. Pore water SO_4_^2−^ decreased with depth, reaching values < 1.5 mM below sediment depths of 11, 15, and 8.5 cm at sites JML, GOF5, and LL3A, respectively. Concentrations of Cl^−^ ranged between ~ 136 and ~ 162 mM in bottom waters, decreased in the sediment until a depth of 10 cm, after which they remained constant at values ranging from ~ 115 to ~ 144 mM (Fig. S4A). Depth profiles of SO_4_^2−^/Cl^−^ indicate that only a small part of the decrease in SO_4_^2−^ with depth is due to a change in bottom water salinity (Fig. S4B).

### Benthic fluxes

**Fig. 5:**
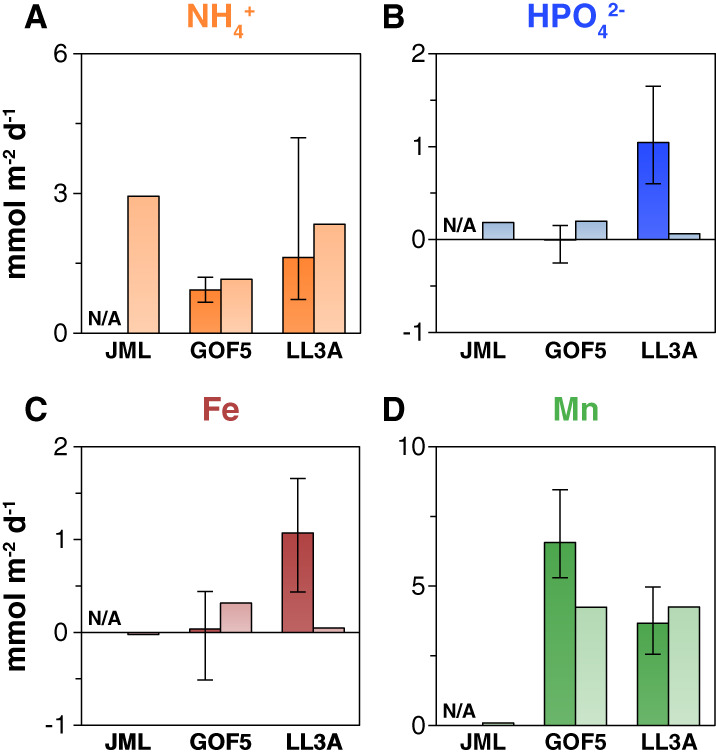
Benthic fluxes of key solutes in mmol^−2^ d^−1^: (**A**) NH_4_^+^, (**B**) HPO_4_^2−^, (**C**) Fe, and **(D)** Mn. Darker colors with minimum and maximum bars represent in‐situ fluxes that were obtained using the benthic lander, whereas the lighter colors represent diffusive fluxes that were calculated from porewater profiles. N/A, not available.

In situ benthic fluxes of dissolved NH_4_^+^, HPO_4_^2−^, and Fe were typically higher at site LL3A when compared to GOF5, with average fluxes at the former site of 1.6, 1.0, and 1.0 mmol m^−2^ d^−1^, respectively (Fig. 5A–C; Figs. S5, S6). Manganese in situ fluxes at sites GOF5 and LL3A were much higher than in situ fluxes of Fe (with values for Mn of 6.6 and 3.7 mmol m^−2^ d^−1^, respectively; Fig. 5D). While in situ benthic fluxes for NH_4_^+^ and dissolved Mn were similar to calculated diffusive fluxes (Fig. 5A,D; Table S2), this was not the case for HPO_4_^2−^ and Fe (Fig. 5B,C).

### Sediment geochemistry

**Fig. 6:**
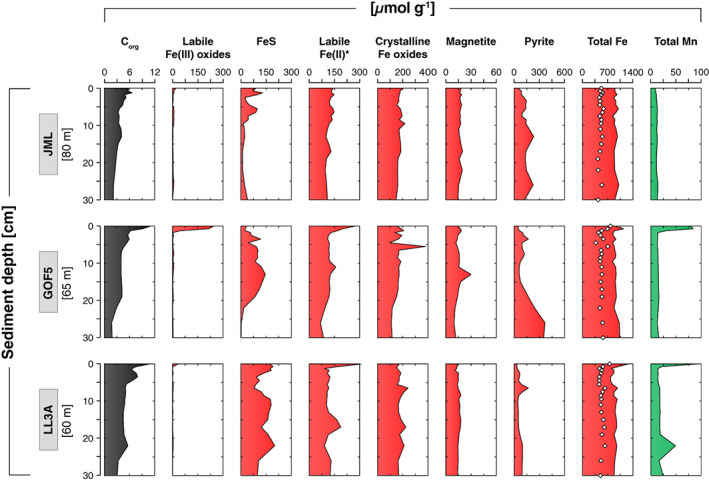
Solid‐phase depth profiles of C_org_ (black), labile Fe(III) oxides, FeS, labile Fe(II) *corrected for FeS, crystalline Fe oxides, magnetite, pyrite, total Fe (red), and total Mn (green) at sites JML, GOF5, and LL3A in June 2016. The white diamonds represent the total amount Fe extracted in the sequential procedure.

Average sediment accumulation rates ranged from 0.4 to 1.5 cm yr^−1^ (Table 1; Figs. S7, S8). Sediment C_org_ was highest at the top and decreased with sediment depth at all sites (Fig. 6). Similar depth trends were observed in sediment N_org_, with C_org_/N_org_ varying around ~ 9 mol mol^−1^ (Fig. S9A). Ratios of S/C_org_ increased with sediment depth and were mostly < 0.4, except for several samples from the SO_4_^2−^ depletion zone (Fig. S9B). Small surface enrichments of Fe oxides were observed at sites JML and LL3A (18 and 36 *μ*mol g^−1^, respectively), whereas site GOF5 was characterized by a strong surface enrichment of Fe oxides (250 *μ*mol g^−1^; Fig 6). Relatively high surface FeS levels were found at sites JML and LL3A (90 and 175 *μ*mol g^−1^, respectively), whereas FeS was depleted in the surface sediment at site GOF5 (34 *μ*mol g^−1^). At GOF5, FeS increased with depth within the first 15 cm of the sediment followed by a decrease, which coincided with a strong increase in sedimentary pyrite. Labile Fe(II) corrected for FeS exhibited strong surface enrichments at sites GOF5 and LL3A (~ 300 *μ*mol g^−1^). Crystalline Fe oxides and magnetite showed no trend with sediment depth at all sites. Total extractable Fe represented ~ 40–70% of the total Fe pool. Total Mn was low at site JML, whereas surface enrichments in total Mn were observed at sites GOF5 and LL3A (85 and 96 *μ*mol g^−1^, respectively). Weight ratios of Mn/Al were always below 0.1 at all sites (Fig. S10).

Profiles of DOS and DOP were variable but generally increased with sediment depth at all sites with most values remaining below 0.42 (Fig. S11A,B). Sediment Py‐Fe(II) increased with sediment depth to values of 0.26–0.64 at 30 cm depth (Fig. S11C). Sid‐Fe(II) decreased to values of 0.35 to 0.45 over the same depth interval (Fig. S11D). Fe/Al were highest near the sediment–water interface at sites GOF5 and LL3A, reaching values of ~ 1 and 1.2, respectively, but varied between ~ 0.6 and 0.8 at depth and throughout the sediment interval sampled at site JML (Fig. S11E). Sediment FeHR/total Fe varied between 0.36 and 0.82 with most values close to 0.5 (Fig. S11F).

**Fig. 7:**
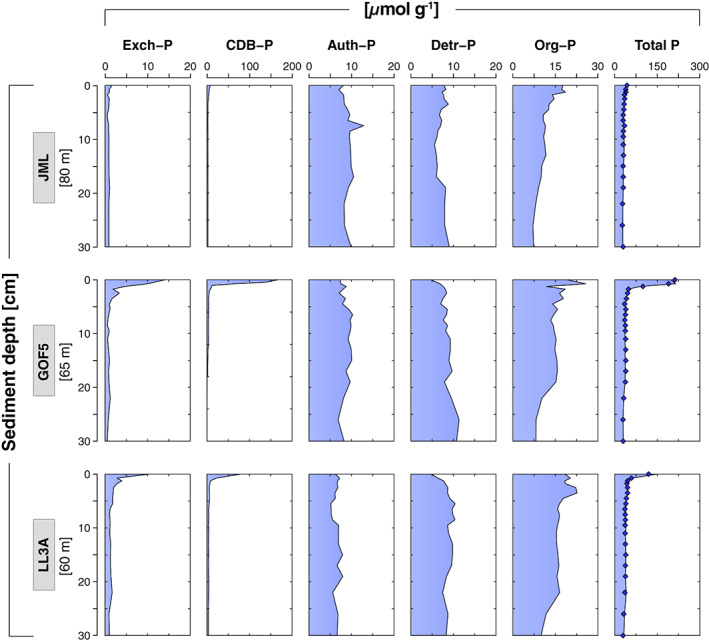
Solid‐phase depth profiles of exchangeable‐P, CDB‐P, authigenic‐P, detrital‐P, organic‐P, and total P sites JML, GOF5, and LL3A in June 2016. Dark blue diamonds represent the total amount of P extracted in the sequential procedure.

Exchangeable‐P and CDB‐P, hereafter referred to as metal bound P, were low at site JML, whereas sites GOF5 and LL3A exhibited strong surface enrichments of ~ 200 and ~ 100 *μ*mol g^−1^, respectively (Fig. 7). Authigenic‐P and detrital‐P showed no clear trend with depth in the sediment at all sites. Organic‐P was highest near the surface and decreased with sediment depth at all sites. Total P burial rates ranged from 43 to 147 mmol m^−2^ yr^−1^ (Table 1). The organic‐P fraction accounted for 27% to 37% of total P burial.

### High‐resolution elemental mapping of surface sediment

**Fig. 8:**
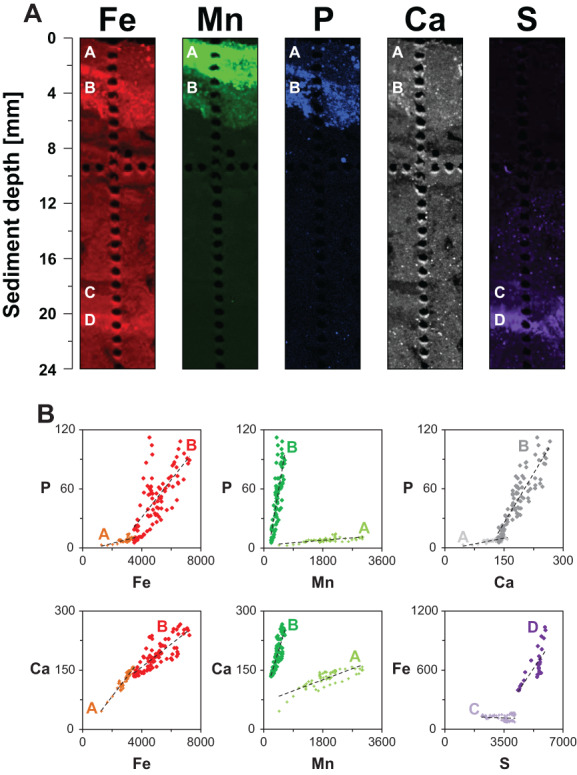
(**A**) Desktop *μ*XRF maps of Fe (red), Mn (green), P (blue), Ca (gray), and S (purple) of the top 24 mm of the (epoxy embedded) surface sediment at site GOF5. Zone A, B, C, and D represent the sediment depth intervals that were investigated for elemental correlations. (**B**) Correlations between P and Fe, P and Mn, and P and Ca, and between Ca and Fe, Ca and Mn, and Fe and S in relevant zones. The elemental ratios and R^2^ of these linear correlations are given in Table S3.

High‐resolution desktop *μ*XRF mapping of Fe, Mn, P, Ca, and S revealed a surface sediment layer highly enriched in Mn (zone A; 0–2 mm; Fig. 8A). Below this layer, a well‐defined layer highly enriched in Fe was observed, in which the spatial distribution of Fe and Mn closely resembled the distribution of P (zone B; 2–6 mm; Fig. 8A). Counts of Fe and P in zone A and zone B were linearly correlated, although the Fe : P ratios differed (290 : 1 and 75 : 1, respectively; Table S3 and Fig. 8B). Counts of Mn and P were also linearly correlated and were characterized by even larger differences in Mn : P ratios between zone A and zone B (315 : 1 and 5 : 1, respectively; Table S3 and Fig. 8B). This implies that relatively more P was associated with Fe and Mn in zone B compared to zone A. Deeper in the sediment (zone C; 18–20 mm) total S increased and further down (zone D; 20–22 mm), the enrichment in S coincided with an enrichment in Fe (Fig. 8A).

### Synchrotron‐based Fe analysis of the sediment

**Fig. 9:**
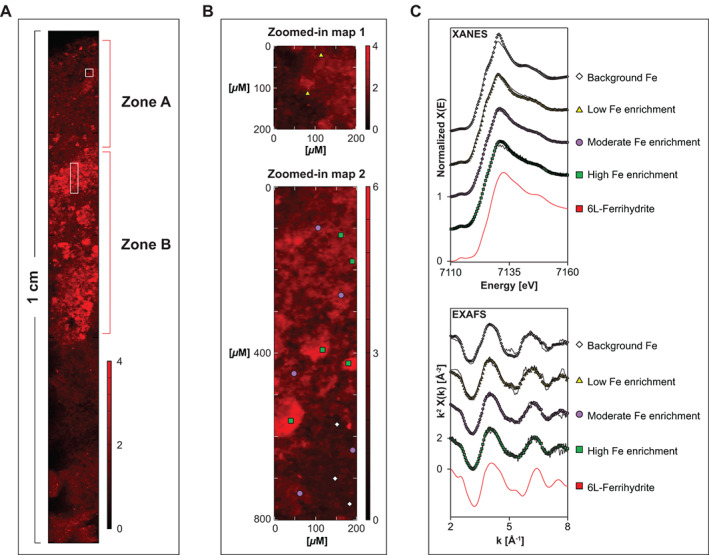
Synchrotron‐based Fe analyses for epoxy embedded surface sediment at site GOF5: (**A**) high‐resolution *μ*XRF map of the first 10 mm of the surface sediment. The white square and rectangle represent areas that were analyzed in further detail (**B**). These maps are shown in true vertical orientation, and the colors accentuate the relative counts adjusted for brightness and contrast to highlight the Fe enrichments in the sediment. The labels indicate the spots that were further subjected to XANES and EXAFS analysis: gray diamonds = background Fe; yellow triangles = low Fe enrichment; purple circles = moderate Fe enrichment; green squares = high Fe enrichment. (**C**) Normalized XANES and *k* ^2^ weighted EXAFS spectra of Fe. The points are measured data; the lines reflect the results from LCF (Fig. S12).

The Fe XANES and EXAFS spectra collected from the Fe enriched spots in zone A and zone B (Fig. 9A,B) were similar to each other (Fig. 9C). However, the spectra showed subtle differences related to the relative Fe enrichment (Table S1). For XANES spectra, increased Fe contents led to a shift of the edge position toward the right at a higher energy and a broadening of the maximum absorption peak. For EXAFS spectra, increased Fe contents led to major oscillations within the range of 3 to 7 Å^−1^ shifted toward higher *k* values (Fig. 9C). The similarity in the spectra can be attributed to the relatively large analyzed volume due to the “infinite thickness” of the sample and a penetration depth beyond 100 *μ*m at the used X‐ray energy. All Fe enrichments are smaller than 100 *μ*m (Fig. 9B) implying that the obtained spectra include a signal from Fe in particles outside the enrichments, which is assumed to be detrital Fe. An average Fe XANES spectrum retrieved from spots in the sediment containing very little Fe (hereafter referred to as background Fe and assumed to reflect detrital Fe), could be best reproduced by LCF of XANES spectra from biotite and illite (Supplementary Information 1.16; Fig. S12).

To reveal information on the Fe enrichments, the spectra collected at all spots were subjected to LCF by combining the spectrum from background Fe with that of other reactive Fe phases (Fig. S12). Only when combining the spectra of background Fe with that of 6L‐ferrihydrite, consistent results were obtained when applied to XANES (energy range 7103–7183 eV) and *k* ^2^ weighted EXAFS spectra (*k*‐range 2–8 Å^−1^). The fitting results indicate an increased contribution of 6L‐ferrihydrite with an increase in the Fe content (Table 2).

**Table 2:** Composition of Fe minerals at site GOF5.

Spectrum	Fe XANES		Fe EXAFS	
	Background Fe (%)	6L‐ferrihydrite (%)	Background Fe (%)	6L‐ferrihydrite (%)
Low Fe enrichment	97±1	3±1	84±4	16±4
Moderate Fe enrichment	65±1	35±1	64±3	36±3
High Fe enrichment	53±2	47±2	52±3	48±3

### Synchrotron‐based Mn analysis of the sediment

**Fig. 10:**
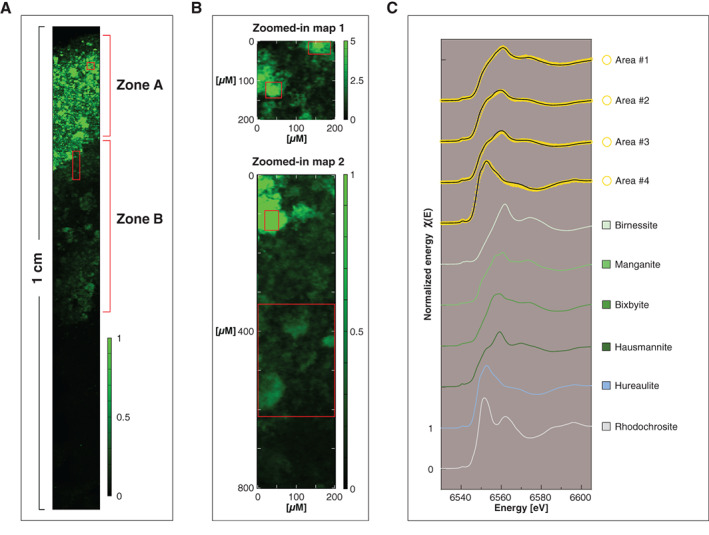
Synchrotron‐based Mn analyses for epoxy embedded surface sediment at site GOF5: (**A**) high‐resolution *μ*XRF map of the first 10 mm of the surface sediment. The red rectangles represent areas that were analyzed in further detail (**B**). These maps are shown in true vertical orientation, and the colors accentuate the relative counts adjusted for brightness and contrast to highlight the Fe enrichments in the sediment. The red rectangles indicate the areas that were further subjected to XANES analysis. (**C**) Normalized Mn XANES spectra.

Principal component analysis of all collected Mn XANES spectra in combination with LCF suggested that using the spectra of six reference Mn minerals, birnessite, hausmannite, bixbyite, manganite, Mn(II) phosphate (hureaulite) and rhodochrosite was the most consistent approach to reproduce all the spectra (Supplementary Information 1.17). The Mn XANES spectra retrieved from the Mn‐rich areas in the upper part of the surface sediment (area #1–3; Fig. 10) systematically deviated from those in the Fe‐enriched zone (area #4) whose edge position and white line was located at lower energies than in the spectra above. LCF of the XANES spectra allowed to reproduce the edge position and the adsorption maxima at the various spots which, both, reflect the oxidation state of Mn. For detailed analysis of the Mn mineralogy, EXAFS spectra would be required. Hence, the interpretation of the LCF results was limited to distinguish Mn oxides with various oxidation states from Mn(II) carbonates and phosphates. According to the LCF of the Mn XANES spectra, Mn oxides (area # 1–3) accounted for ~ 74–87% of total Mn, with Mn(II) phosphates and carbonates accounting for the remaining ~ 5–26% (Table 3). The XANES spectra for area #4 closely resemble that of Mn(II) phosphates and LCF suggest that Mn (II) phosphates account for about ~ 97% of total Mn, while Mn(II) carbonate was absent (Table 3).

**Table 3:** Composition of Mn minerals at site GOF5.

Spectra	Mn oxides (%)	Mn(II) phosphates (%)	Rhodochrosite (%)
Area #1	87±14	5±3	8±8
Area #2	73±12	24±2	3±7
Area #3	74±14	26±3	
Area #4	3±0	97±3	

No values = contribution < 1%.

## Discussion

### Environmental setting and benthic release of Fe, Mn, and P in the Gulf of Finland

Eutrophication of the Baltic Sea has led to the expansion of areas with organic‐rich sediments and bottom waters that are low in O_2_ (Diaz and Rosenberg 2008). Sediment C_org_/N_org_ ratios of ~ 9 (Fig. S9A) are relatively close to the Redfield ratio of 6.6, supporting an important contribution of marine organic matter. The sediment S/C_org_ ratios of < 0.4 (Fig. S9B) reflect deposition in a brackish environment (< 0.5; Berner and Raiswell 1984). The ranges of Sid‐Fe(II) (0.35–0.45) and Pyr‐Fe(II) (0.26–0.64) at 30 cm depth (Fig. S11C,D) indicate a relatively large proportion of nonsulfidized Fe(II), which is also typical for a setting with brackish bottom waters (Aller et al. 2004). The DOS and DOP values are typically < 0.42 (Fig. S11A,B) and hence fall within the range for oxic and dysoxic settings (threshold of 0.45) as defined in Raiswell et al. (2018). The Fe/Al and FeHR/total Fe ratios cover a wide range (0.6–0.8 and 0.36–0.82, respectively; Fig. S11E,F) and mostly fall within the range for anoxic bottom waters (thresholds of 0.66 and 0.38, respectively; Raiswell et al. 2018).

**Fig. 11:**
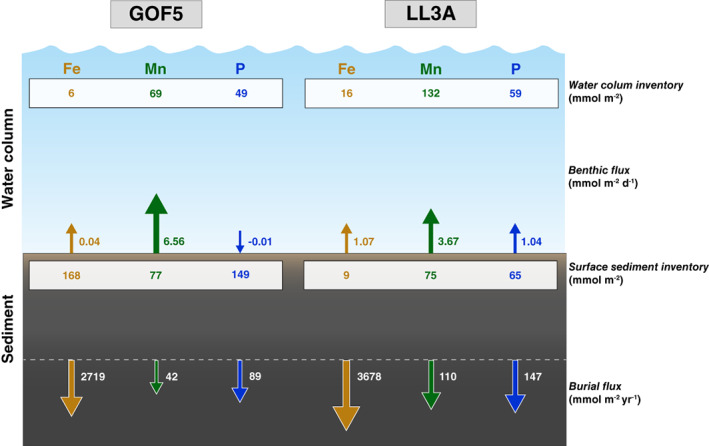
Inventories in the water column and surface sediment, benthic release and burial fluxes for Fe, Mn, and P at sites GOF5 and LL3A. Note the different units for the benthic release and the burial flux of Fe, Mn, and P (mmol m^−2^ d^−1^ vs. mmol m^−2^ yr^−1^).

The lack of O_2_ has led to high concentrations of dissolved HPO_4_^2−^ and dissolved and/or particulate Fe and Mn in the water column of the deep basins of the Baltic Sea (Gotland and Landsort Deep) due to less efficient sequestration in the sediment (Turnewitsch and Pohl 2010). Here, we report high concentrations of total dissolvable Fe and Mn and dissolved HPO_4_^2−^ (1500 nM, 15,000 nM, and 3 *μ*M, respectively) in the bottom waters of the Gulf of Finland (Fig. 2). Combined with high benthic fluxes of dissolved Fe, Mn, and HPO_4_^2−^ at sites GOF5 and LL3A (Fig. 5; Table S2), this implies that release from the sediment contributes to the enrichment of Fe and Mn in the bottom water at seasonally hypoxic sites (Fig. 11; Figs. S13A, S14A). This is confirmed by the elevated Fe/Al and Mn/Al ratios of the particulate matter in the bottom water, which are much higher than those in the sediment (Fe/Al: 20–165 vs. 0.6–1.2, Mn/Al:167–222 vs. 0.006–0.01; Figs. S2, S10, S11E), and typical background concentrations in the Baltic Sea region (Fe/Al: 0.5 and Mn/Al: 0.006–0.01; Lenz et al. 2015*b*). This supports our interpretation of formation of Fe and Mn oxides in the water column.

The form of the Fe and Mn in the bottom water at our study sites depends on its redox state as shown in a parallel study on Fe and Mn dynamics in the Baltic Sea (Lenstra et al. 2021). At site JML, where bottom waters were sulfidic, Fe was likely mainly present as FeS, whereas Mn was present in dissolved form only. This is in accordance with results of previous analyses that the lower (sulfidic) part of the water column in the Baltic Sea is supersaturated with respect to FeS (Dyrssen and Kremling 1990). At sites GOF5 and LL3A, where waters were low in O_2_ but not sulfidic, Fe was mainly present in particulate form as poorly crystalline Fe oxides, whereas dissolved Mn dominated the total dissolvable Mn pool. Nevertheless, there was still a substantial amount of particulate Mn in the bottom water (~ 1600–3100 nM) and we show here that at these latter sites, this particulate Mn is predominantly present in the form of Mn oxides (Fig. 3).

The geochemistry of the surface sediment at sites GOF5 and LL3A differed distinctly from that of site JML, with only the former being characterized by enrichments in Fe and Mn oxides and associated P (Figs. 6, 7) and sharp peaks in pore water Fe and Mn near the sediment–water interface (Fig. 4). Sedimentary P in the surface sediment at sites GOF5 and LL3A was predominantly bound to Fe and Mn minerals (Fig. S15A). At site JML, most of the P in the surface sediment (0–2 cm) was associated with organic matter.

The above suggests there is potential for a continuous cycle of release of dissolved Fe and/or Mn from the sediment, formation of oxides above the thin hypoxic layer in the water column and subsequent deposition of metal oxides, as described for Mn for other seasonally hypoxic coastal systems (Adelson et al. 2001; Sulu‐Gambari et al. 2017). At site GOF5, the high benthic flux of Mn (6.6 mmol m^−2^ d^−1^) and nearly equal amounts of Mn in the water column and surface sediment (69 and 77 mmol m^−2^; Fig. 11; Fig. S13) indicate a turnover time of 12 and 10 d, respectively. This points toward strong refluxing of Mn at this site.

The higher water column concentrations and benthic fluxes of Mn compared to those of Fe at sites GOF5 and LL3A can be explained by their contrasting geochemical dynamics. When bottom water O_2_ is low, dissolved Mn can more easily escape to the overlying water than dissolved Fe (Burdige 1993). Contributing factors include the slower oxidation kinetics of dissolved Mn with O_2_, which is mainly a microbially mediated process, and the less efficient sequestration of Mn in mineral form when compared to Fe (Krauskopf 1955; Burdige 1993). This difference in sequestration is also apparent from the sediment Fe and Mn contents below the surface sediment at all sites: while Fe sulfides account for a significant fraction of reactive Fe (Fig. 6), sediment Mn contents are very low, which is often the case in organic rich deposits where Mn is mainly associated with a background of clays and/or carbonates (Lenz et al. 2014). In the following sections, we will focus on the potential role of cable bacteria activity in determining the formation of Fe and Mn oxides, their preservation in the sediment and P sequestration in Gulf of Finland sediments.

### Potential for diagenetic Fe and Mn mineral formation induced by cable bacteria

Bottom water O_2_ availability acts as a key control on cable bacteria activity (Burdorf et al. 2018) and may have induced the removal of ΣH_2_S and formation of Fe oxides and preservation of Mn oxides in the surface sediment at sites GOF5 and LL3A in spring (Figs. 4, 6). The impact of *Marenzelleria* on the formation of these Fe oxides was likely limited, since much higher population densities than observed at these sites (> 3000 ind. m^−2^ vs. 1200 ind. m^−2^; Hermans et al. 2019) are necessary to significantly promote the formation of Fe oxides (Norkko et al. 2012). However, meiofauna can be abundant in Baltic Sea sediment (Broman et al. 2020) and could thus impact the cycling of Fe and Mn, by deepening O_2_ penetration. Meiofauna can possibly coexist with cable bacteria (Bonaglia et al. 2020). At site JML, the lack of O_2_ will have hindered cable bacteria activity (Burdorf et al. 2018) and the presence of ΣH_2_S in the bottom water during part of the year will have prevented the formation and preservation of Fe and Mn oxides.

The potential for recent impact of cable bacteria activity on surface sediment geochemistry was largest at site GOF5 (Figs. 6, 7) and is supported by visual observations of the surface sediment at the time of sampling. At this site, an orange Fe oxide layer (oxic zone), was observed above a gray FeS depleted layer (suboxic zone) and a black layer (sulfidic zone; Fig. S16). This color stratigraphy is typical for sediments that have been geochemically altered by cable bacteria (Nielsen and Risgaard‐Petersen 2015; Sulu‐Gambari et al. 2016*a*; Hermans et al. 2020), although may not always necessarily be exclusively tied to activity of cable bacteria. Sites LL3A and JML (Fig. 6; Fig. S17) did not exhibit these characteristic features, therefore only the samples for site GOF5 were studied in detail for their mineralogy. The lack of such features at site LL3A is remarkable because of the higher number of cable bacteria compared to site GOF5. As discussed previously by Hermans et al. (2019), this may be explained by the higher SO_4_^2−^ reduction rate and hence faster conversion of the Fe oxides that were formed earlier in spring to Fe sulfides at site LL3A when compared to site GOF5.

The Fe oxides, which possibly formed by the activity of cable bacteria in spring at site GOF5, were focused within a thin layer near the sediment–water interface (zone B; 2–5 mm depth; Fig. 8A). The reactive Fe in this layer predominantly consisted of poorly crystalline Fe (III) oxides (Fig. 9; Table 2). The size of the deficit in FeS in the upper 2 cm of the sediment is equal to that of the enrichment in Fe oxides (both 0.17 mol m^−2^), which Hermans et al. (2019) attributed to full conversion of FeS to Fe oxides as a consequence of pore water acidification by cable bacteria in spring, (Fig. 6; Fig. S13B). For comparison, the amount of Fe in the water column at the time of sampling was 0.006 mol m^−2^ (Fig. 11; Fig. S13A).

Directly above the Fe‐rich layer and below the sediment–water interface (zone A; 0–2 mm depth), the sediment was highly enriched in Mn oxides (Figs. 8A, 10; Table 3). Some of these Mn oxides may have formed diagenetically in spring from upward diffusing dissolved Mn. Given the position of the redox cline in the water column, Mn oxides likely did not form in the sediment at the time of sampling (Fig. 1), and deposition of suspended matter from the water column was likely the main source of Mn in the surface sediment.

Underneath the Mn oxide rich layer, thus in the same zone as the Fe oxide enriched layer (zone B; 2–5 mm depth; Fig. 8A), a moderate enrichment in Mn was observed, mainly consisting of Mn(II) phosphates (Fig. 10; Table 3). The formation of Mn(II) phosphates is likely favored by high concentrations of dissolved Mn and HPO_4_^2−^ and low levels of ΣH_2_S. Such conditions were present in the surface sediment at site GOF5 (Fig. 4).

The relative contributions of the Fe and Mn oxides and Mn(II) phosphates in sequestering P in the surface sediment at site GOF5 was estimated (Table 4) by combining the sequential extraction and *μ*XRF data. The total amount of metal bound P (Ex‐P + CDB‐P) in the top 5 mm of the surface sediment was 0.048 mol m^−2^ (Fig. 7) of which 10% (0.005 mol m^−2^) was located in zone A and 90% (0.043 mol m^−2^) in zone B (Fig. S18). The total amount of Fe integrated over the top 5 mm of the surface sediment at site GOF5 amounted to 0.24 mol m^−2^ (Fig. 6). Approximately, 29% (0.07 mol m^−2^) of total Fe was located in zone A (0–2 mm) and 71% (0.17 mol m^−2^) in zone B (2–5 mm; Fig. S18). The relative contribution of Fe to P sequestration in zone A was likely negligible, since illite and biotite are both relatively not as efficient in sequestering P compared to Fe oxides (Edzwald et al. 1976; Gunnars et al. 2002). In contrast, the Fe minerals in zone B, likely did contribute to P sequestration. Approximately 38% of the total Fe in zone B consisted of poorly crystalline Fe oxides (Table 2). Assuming a 2 to 1 ratio of Fe to P (Gunnars et al. 2002; Table 4), these Fe oxides (0.066 mol m^−2^) could potentially sequester 0.033 mol P m^−2^.

**Table 4:** Relative contribution of Fe and minerals to P sequestration in the surface sediment at site GOF5.

Layer	Fe oxides (%)	Mn oxides (%)	Mn(II) phosphates
0–2 mm (zone A)	0	10	0
2–5 mm (zone B)	68	0	22

Layer	Fe oxides (mmol m^−2^)	Mn oxides (mmol m^−2^)	Mn(II) phosphates (mmol m^−2^)
0–2 mm (zone A)	0	4.9	0
2–5 mm (zone B)	32.9	0	10.3

The total amount of Mn in the top 5 mm of the surface sediment amounted to 0.021 mol m^−2^. Approximately, 73% (0.015 mol m^−2^) of total Mn was located in zone A (0–2 mm) and about 27% (0.006 mol m^−2^) in zone B (2–5 mm; Fig. S18). Because Fe oxides were not present in zone A, the relative contribution of Mn oxides in this zone with respect to P sequestration was ~ 10%. The Mn(II) phosphates in zone B accounted for the remaining 22% (0.010 mol m^−2^) of P sequestration.

To summarize, P sequestration in the surface sediment of GOF5 occurred in a very narrow zone of the sediment and was predominately associated with Fe oxides (68%), whereas Mn oxides and Mn(II) phosphates both played a smaller role (32%; Table 4). Hermans et al. (2019) previously attributed the pore water acidification and associated FeS dissolution to potential cable bacteria activity in spring, pointing toward a diagenetic source of Fe oxides in the surface sediment at site GOF5. Deposition of suspended matter from the water column likely acted as the main source for sedimentary Mn oxides.

### Implications for water quality in seasonally hypoxic systems

In the Gulf of Finland, release of HPO_4_^2−^ from metal oxides and organic matter during periods with low bottom water O_2_ have been suggested to control the highly varying concentrations of HPO_4_^2−^ in the bottom water (Pitkänen et al. 2001). Water column monitoring data (SMHI) reveals that bottom water O_2_ is typically inversely correlated with HPO_4_^2−^ (Fig. S19; Viktorsson et al. 2012). At the time of sampling bottom water O_2_ was low, whereas benthic fluxes of HPO_4_^2−^ were relatively high, ranging up to 1.65 mmol m^−2^ d^−1^ (Table 1; Fig. 5B). Similar high fluxes of HPO_4_^2−^ were observed previously at other sites in the Gulf of Finland upon reductive dissolution of metal oxides in the sediment (Pitkänen et al. 2001; Lehtoranta 2003; Viktorsson et al. 2012). Pore water NH_4_^+^/HPO_4_^2−^ ratios at our sites are mostly < 4 (Fig. S20), which is much lower than the Redfield N/P ratio of 16, hence supporting release of HPO_4_^2−^ from metal oxides. The Fe and Mn minerals formed in spring may temporarily buffer the release of HPO_4_^2−^ in summer. Recycled Fe and Mn oxides depositing from the water column potentially could also contribute to this buffer. We find that this refluxing process is most pronounced for Mn. Cable bacteria can amplify such refluxing (Sulu‐Gambari et al. 2017), since their activity typically removes ΣH_2_S in the surface sediment. This allows dissolved Fe and Mn to escape more easily from the sediment into the water column where it can precipitate as Fe and Mn oxides upon contact with O_2_, and return to the sediment through deposition.

At site GOF5, bottom water records indicate that the layer of poorly crystalline Fe oxides in the surface sediment likely is not completely removed during hypoxia (Fig. S19). Complete reductive dissolution of this Fe oxide layer would give an expected increase of 7.4 *μ*M HPO_4_^2−^, assuming benthic release affects the lower 20 m of the water column (Table S4). The maximum increase observed in the bottom water HPO_4_^2−^ at site GOF5, was only ~ 4 *μ*M during peak bottom water anoxia (Fig. S19). At site LL3A, most of the poorly crystalline Fe oxides and associated P formed in spring likely already underwent reductive dissolution at the time of sampling, leading to a relatively low content of these Fe oxides and relatively high FeS content in the surface sediment (Fig. 6). The relatively higher SO_4_^2−^ reduction rate observed at site LL3A (2.7 mmol m^−2^ d^−1^) compared to that of site GOF5 (2 mmol m^−2^ d^−1^; based on measured and calculated rates of SO_4_^2−^; Hermans et al. 2019) could explain this observation. As a consequence of spatial variability in bottom water O_2_ availability and SO_4_^2−^ reduction rates in the seafloor, the results for site GOF5 cannot be generalized for the entire Gulf of Finland (Fig. 11).

Although cable bacteria may enhance the seasonal sequestration of P in the surface sediment, their potential role in permanent P burial in the Gulf of Finland is limited. Despite a stark contrast in bottom water redox conditions and surface sediment geochemistry, all sites exhibit a strong similarity in pore water and solid‐phase geochemistry below 2 cm sediment depth (Figs. 4, 6, 7). At all sites, most of the decrease in sediment P (mostly upper 2 cm) is caused by the reduction of Fe and Mn minerals to which P is bound, and due to the breakdown of organic P. Permanent burial of P is mostly in the form of organic P, detrital P and authigenic P, with only a minor fraction (11–15%) bound to Fe and Mn minerals (Fig. S15B). This distribution in sedimentary P burial pools is in line with previous observations for Gulf of Finland sediments (Lukkari et al. 2009). Rates of P burial in our study (43–147 mmol m^−2^ yr^−1^; Table 1; Fig. 11) also agree with previous estimates for Gulf of Finland sediments (42–139 mmol m^−2^ yr^−1^; Pitkänen 1994; Lukkari et al. 2008; Asmala et al. 2017). Our data suggest that the variations in bottom water redox conditions at our sites and differences in cable bacteria abundance and, hence, likely differences in recent activity, do not impact the mineral forms and burial rate of Fe, Mn, and P in the deeper sediment (Fig. 11; Fig. S15).

## Conclusions

In the Gulf of Finland, the surface sediment geochemistry and benthic exchange of Fe, Mn and P are strongly controlled by changes in bottom water redox conditions and diagenetic processes. The high abundance of cable bacteria and geochemical signature of the sediment suggest that cable bacteria activity may have contributed to formation of enrichments of Fe, Mn and P in the surface sediment. Using micro‐XRF analyses combined with synchrotron‐based X‐ray spectroscopy, we show that the near surface enrichments consist of poorly crystalline Fe oxides, Mn(II) phosphates and Mn oxides. The Fe oxides are focused within a thin layer (3 mm), and are responsible for ~ 68% of the P sequestration in the surface sediment, while Mn minerals account for the remaining ~ 32%. Our results highlight that only a very narrow zone of the sediment is involved in the recycling of Fe, Mn, and P in this brackish, seasonally hypoxic coastal area. While variations in bottom water O_2_ affect the temporal sequestration of Mn and P, it does not impact the permanent burial of Fe, Mn, and P in Gulf of Finland sediments. Further research is required to confirm whether cable bacteria activity indeed promotes the formation of distinct layers enriched in Fe, Mn, and P in Gulf of Finland sediments.

## Conflict of interests

The authors declare that they have no conflicts of interest.

## Supporting information

**Appendix S1:** Supporting information

**Appendix S2:** Supporting information
