# Supplementary material for: Coupled dynamics of iron, manganese, and phosphorus in brackish coastal sediments populated by cable bacteria
Source: Limnol Oceanogr. 2021 May 6;66(7):2611–31. doi: 10.1002/lno.11776 (PMC8360020; doi:10.1002/lno.11776)
Supplement: Supplementary file 1 — Appendix S1: Supporting information [file LNO-66-2611-s001.pdf]

Supporting Information: Coupled dynamics of iron, manganese, and phosphorus in brackish coastal sediments populated by cable bacteria

Martijn Hermans,[1] Marina Astudillo Pascual,[1,3] Thilo Behrends,[1] Wytze K. Lenstra,[1] Daniel J. Conley,[2] Caroline P. Slomp[1]

[1]Department of Earth Sciences (Geochemistry), Faculty of Geosciences, Utrecht University, Utrecht, the Netherlands
[2]Department of Geology, Faculty of Science, Lund University, Lund, Sweden
[3]Department of Biology and Geology, University of Almería, Almería, Spain

1.1 Water Column Temperature, Salinity and Density Depth Profiles

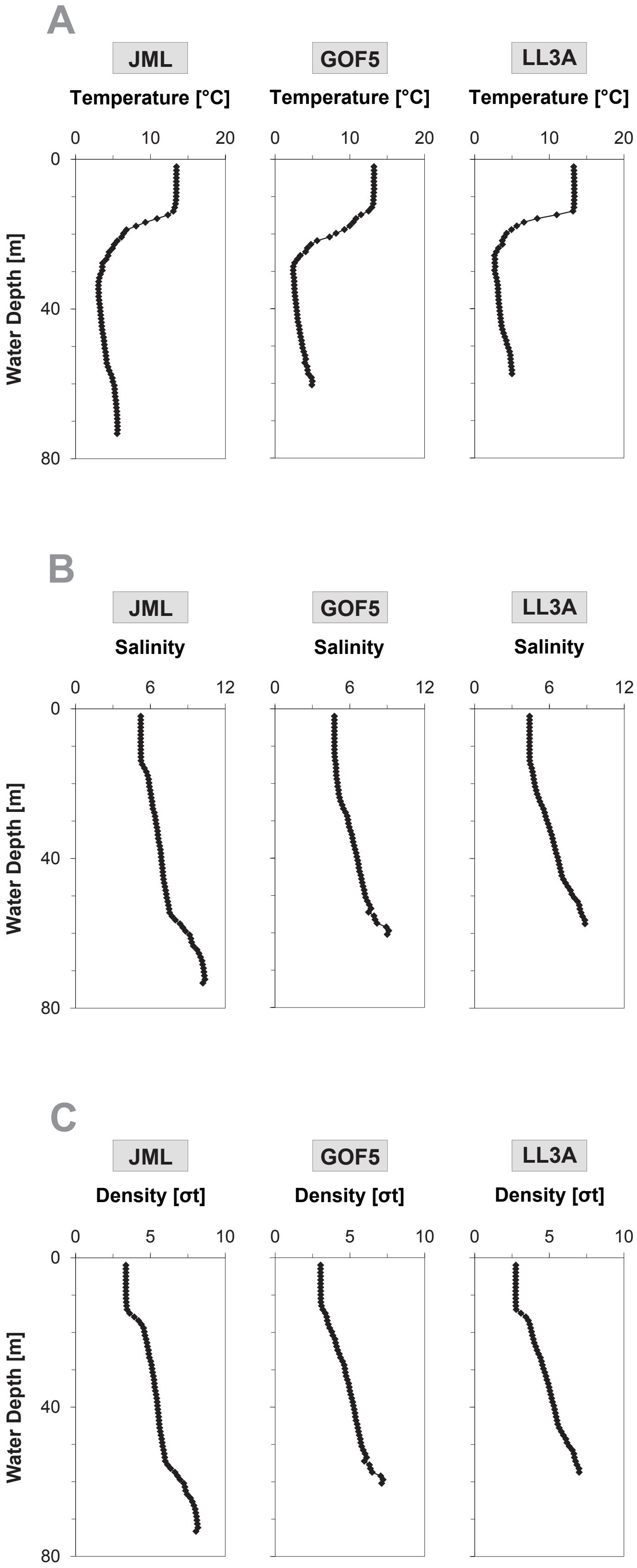

Figure S1. Water column depth profiles of (**A**) Temperature in °C, (**B**) Salinity and (**C**) Density.

1.2 Diffusive Flux Calculations

The diffusive fluxes of dissolved NH_4^+, HPO_4^{2-}, Fe and Mn were calculated using Fick's first law as described in Berner (1980):

$$J = -\phi D_s \cdot \frac{dC}{dz} \quad \text{(S.1)}$$

where J is the diffusive flux [mmol m^{-2} yr^{-1}], ϕ represents the porosity, D_s is the diffusion coefficient [m^2 yr^{-1}], C is the concentration and z represents the sediment depth [m]. The diffusion coefficient was calculated as a function of ambient tortuosity, pressure, salinity and temperature using the R package *marelac* (Soetaert et al. 2010), which implements the constitutive relations described in Boudreau (1997). The concentration gradient between the bottom water and the first sediment depth interval was used for the calculations.

1.3 Iron, Manganese and Phosphorus Burial Calculations

The total Fe, Mn and P burial rates in mmol m^{-2} yr^{-1} were calculated as a function of the sedimentation rate, total Fe, Mn and P in the deeper sediment and the porosity using the following equation:

$$P_{burial} = P_{total} \cdot SR \cdot \rho \cdot (1 - \phi) \cdot 10^4 \quad \text{(S.2)}$$

where, in this case, P_{total} represents the average concentration of total P (mol g^{-1}) in the 20 to 30 cm sediment depth interval, *SR* is the sedimentation rate (cm yr^{-1}), ϕ is the porosity within the same depth interval and ρ represents the density of dry sediment 2.65 g cm^{-3} (Burdige 2006). Total Fe and Mn burial were calculated in a similar way.

1.4 Manganese XAS Analysis of Suspended Matter Samples

Suspended matter samples from the water column from sites GOF5 and LL3A, were analyzed for their Mn content and mineralogy. High-resolution µXRF maps (1.6-2.5 mm^2) were analyzed using a horizontal and vertical step size of 0.5 to 0.75 µm, respectively. Mn XANES spectra were retrieved at 12 to 14 spots that were enriched in Mn. At each spot only one Mn XANES spectrum was collected to avoid photo-induced reduction of Mn during X-ray analyses. Mn XANES spectra, were subjected to component analysis using the Iterative Transformation Factor Analysis (ITFA) software package (Rossberg et al. 2003).

The indicator function from the component analysis revealed that three components are the optimal number for reproducing the Mn XANES spectra of suspended material at the three locations. Spectra from three spots were identified based on the Varimax analysis, each having a high loading on one of the components and only a minor loading on the others. None of the three Mn XANES spectra showed a close resemblance with one of the spectra from reference materials including several Mn oxides, Mn carbonates, Mn phosphates and Mn sulphides. The position of the absorption maximum of all spectra was close to that of Mn in birnessite. The spectra differ, in particular, regarding the shape and position of the edge which is shifted to lower energies compared to birnessite. This indicates that the fraction of Mn with a lower oxidation state than IV (e.g. Mn(II) or Mn(III)) at the analyzed spots is higher than that of Mn in birnessite. Birnessite contains predominately Mn(IV) but can also contain Mn(III) which leads to an excess negative charge in the layers which is compensated by accommodating cations in the interlayer.

Based on the results from the component analysis, the maximum number of spectra for LCF was constrained to three. For LCF, the normalized Mn XANES spectra in the range 6530 to 6580 eV was used. When searching the best combination of three Mn XANES spectra out of a set of spectra from various materials for reproducing the three spectra by LCF, consistently birnessite was selected in combination with hausmannite (Mn_3O_4), Mn(II) phosphates or dissolved Mn^{2+}. The contribution of aqueous Mn^{2+} to one of the spectra was only very minor. Hence, the Mn XANES spectra can be well reproduced when combining the spectra of birnessite, hausmannite and Mn(II)phosphate. The R-factor (sum((fit-data)^2)/sum(data^2)) was on average 0.00167 and always below 0.005.

The quality of the spectra was insufficient for investigating their extended X-ray absorption fine structure (EXAFS). Hence, confirmation of the results from LCF of XANES spectra by investigating the EXAFS was not possible. Furthermore, due to the relatively short energy range, the results of LCF are strongly influenced by the position and shape of the edge. Consequently, the assigned fractions of hausmannite and Mn(II) phosphates might not necessarily reflect the concentrations of these two phases but, instead, account for the presence of Mn(II) and Mn(III) including, for example, also adsorbed Mn(II) or birnessite with a higher Mn(III) content compared to the reference material in the LCF.

1.5 Iron Enrichment Classification

Spots in the synchotron-based μXRF map were classified into three categories based on their total Fe content as reflected in the relative count intensities. (Table S1).

Table S1. Classification of Fe based on relatively count intensity bandwiths.

Classfication	Counts
Low	2 - 3
Moderate	3 - 6
High	6 - 8

1.6 Water Column Depth Profiles of Fe/Al and Mn/Al Ratios

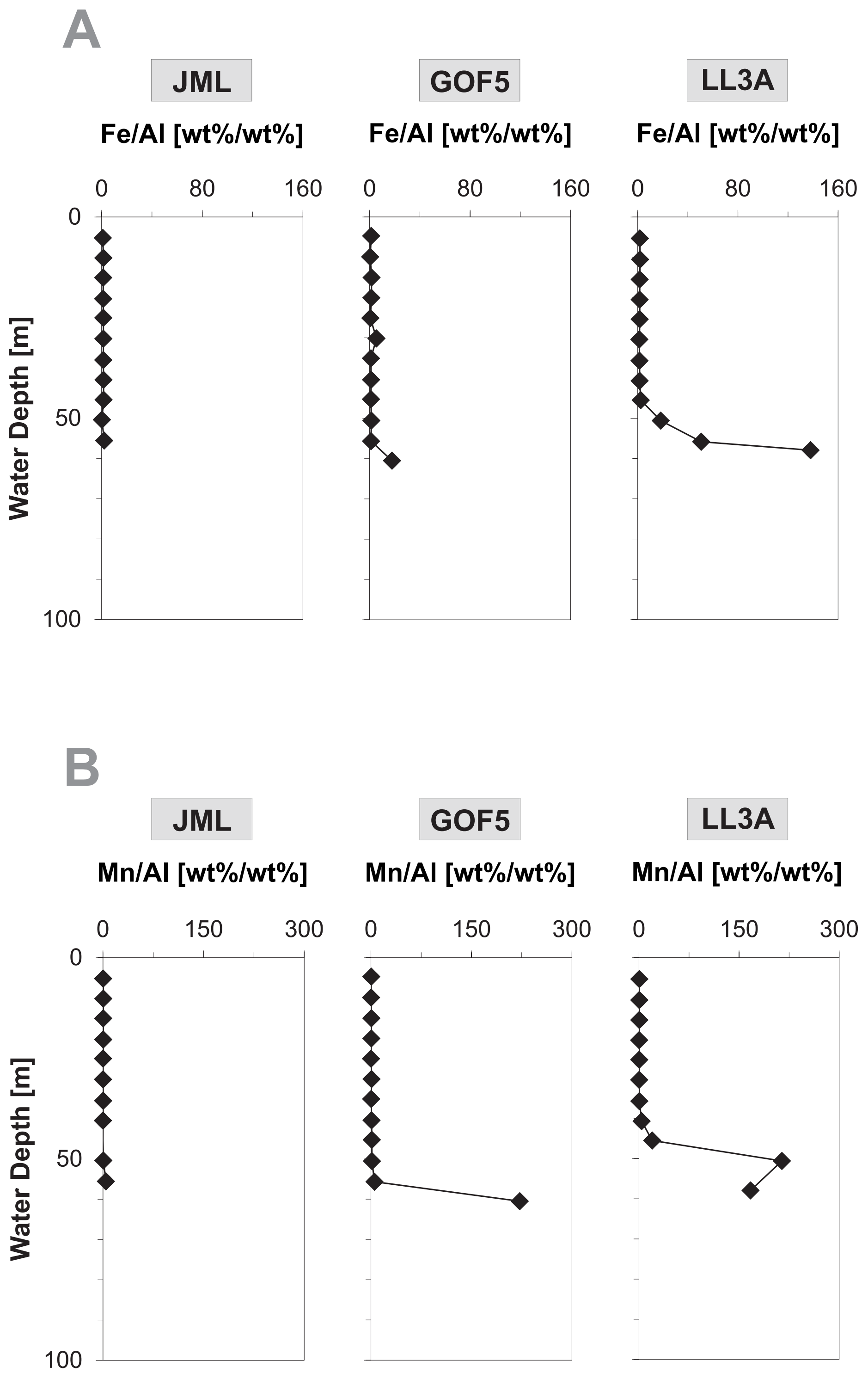

Figure S2. Water column depth profiles of particulate Fe/Al and particulate Mn/Al ratios in wt% $wt\%^{-1}$ for sites JML, GOF5 and LL3A in June 2016.

1.7 High-resolution Maps of Mn in Suspended Matter in the Water Column

At sites GOF5 and LL3A, Mn XANES spectra of suspended matter in the water column were collected from 14 and 12 spots, respectively (Fig. S3).

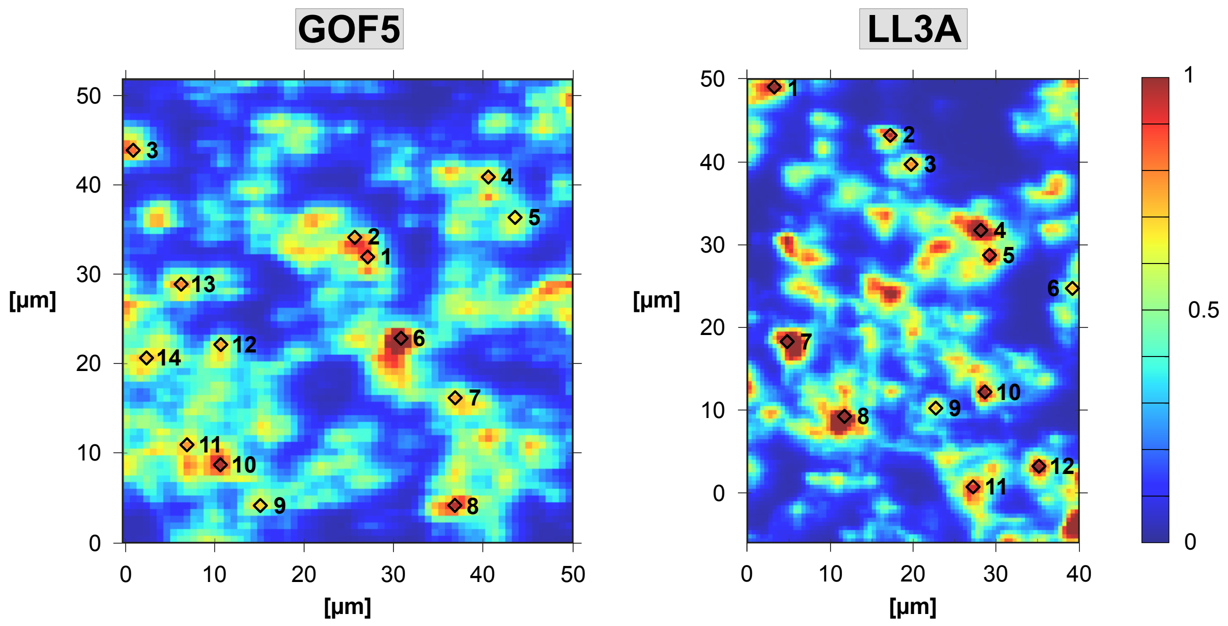

Figure S3. High-resolution synchrotron-based μXRF maps of suspended matter in the water column at sites GOF5 (60 m water depth) and LL3A (58 m water depth). The colors accentuate the relative count intensities adjusted for brightness and contrast to highlight the Mn enrichments. The open black diamonds indicate the Mn enrichments that were further subjected to Mn XANES analysis to identify their mineralogical composition.

1.8 Pore Water Sulfate/Chloride Depth Ratio

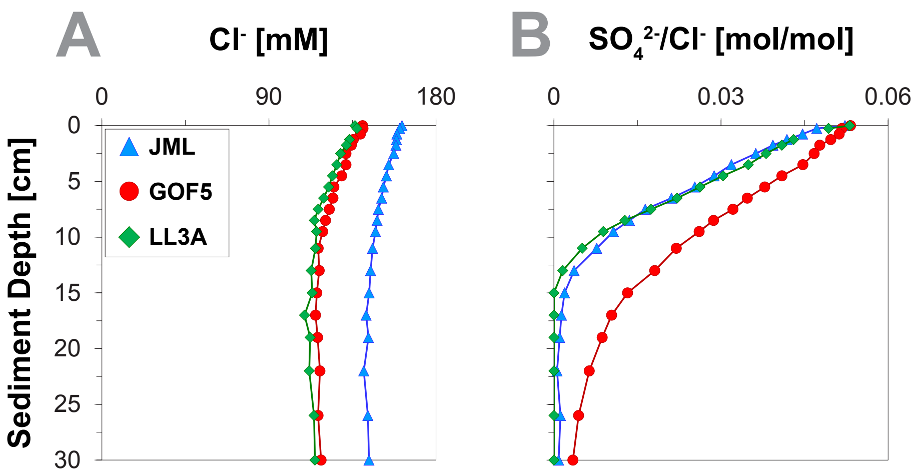

Figure S4. Pore water chloride profiles (A) and sulfate/chloride ratio (B) for sites JML, GOF5 and LL3A in June 2016.

1.9 Benthic Fluxes of Dissolved NH_4^+, HPO_4^{2-}, Fe and Mn

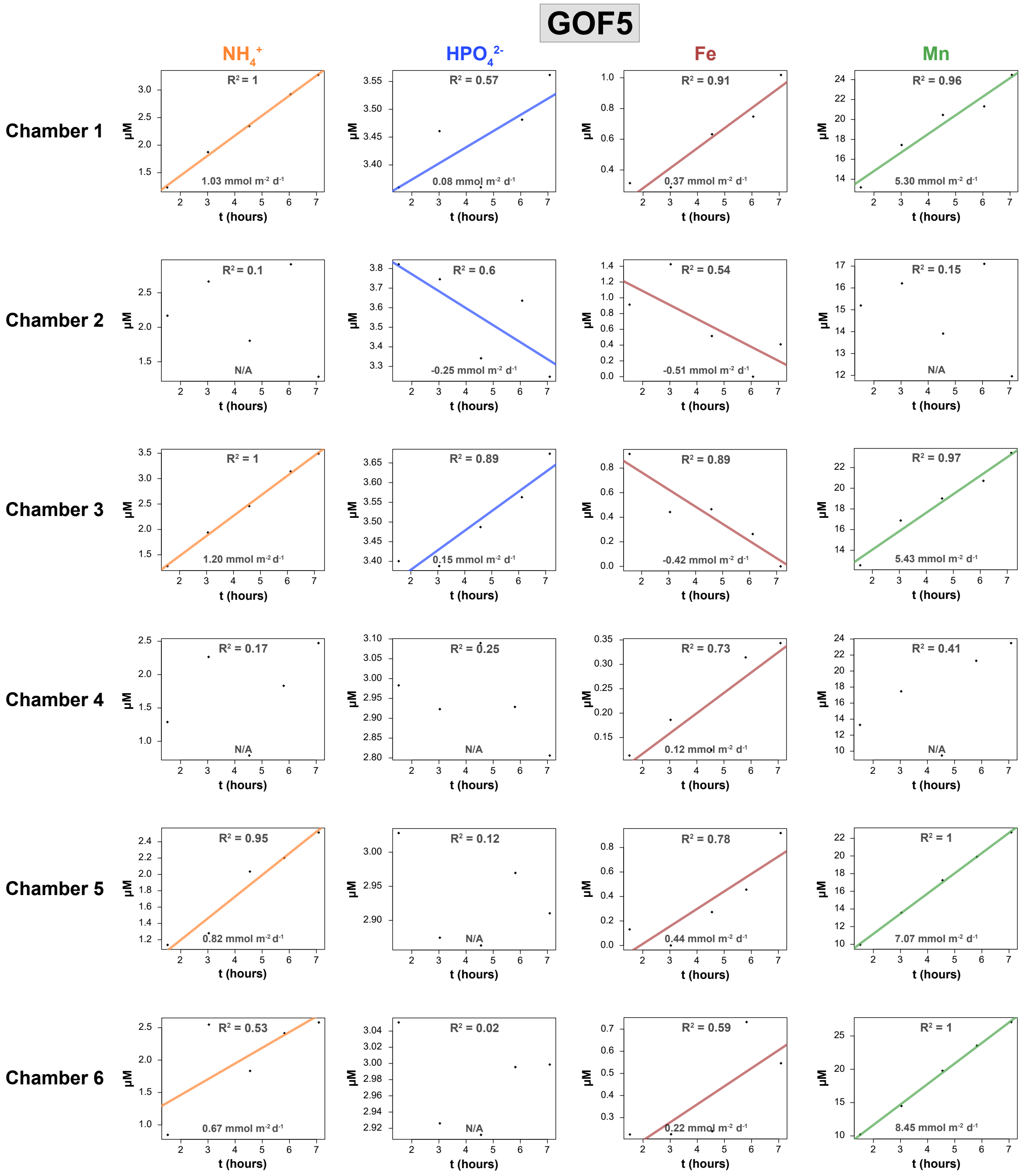

Figure S5. Concentrations of dissolved NH_4^+, HPO_4^{2-}, Fe and Mn derived from six chambers of a benthic lander incubation at site GOF5 in June 2016. The benthic fluxes were determined from the linear correlation and are expressed in mmol m^{-2} d^{-1}. Only linear gradients with an R^2 >0.5 were used for the calculation of the benthic fluxes. N/A = not available (R^2 <0.5).

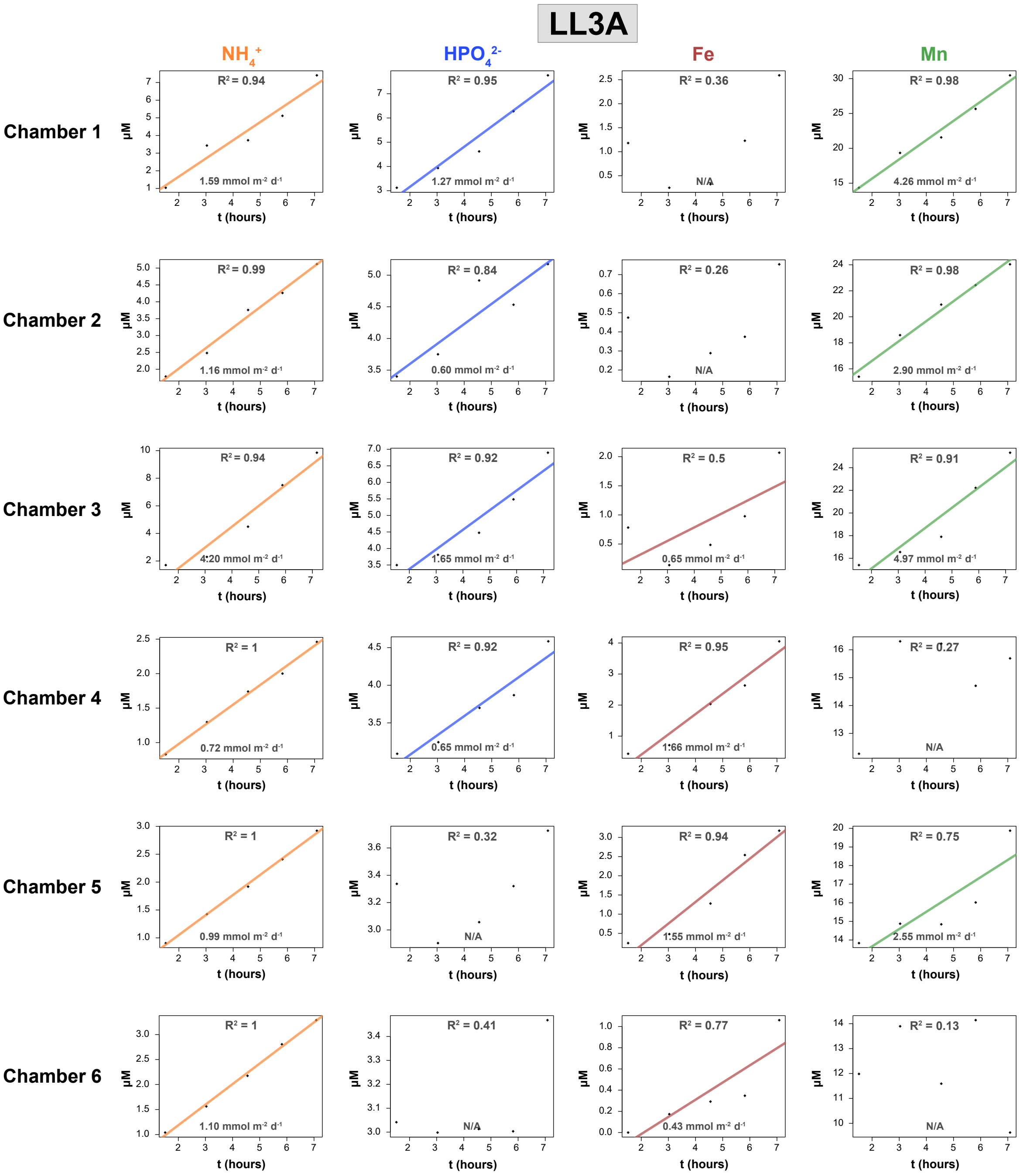

Figure S6. Concentrations of dissolved NH_4^+, HPO_4^{2-}, Fe and Mn derived from six chambers of a benthic lander incubation at site LL3A . The benthic fluxes were determined from the linear correlation and are expressed in mmol m^{-2} d^{-1}. Only linear gradients with an R^2 >0.5 were used for the calculation of the benthic fluxes. N/A = not available (R^2 <0.5).

Table S2. Benthic fluxes of dissolved NH_4^+, HPO_4^{2-}, Fe and Mn in mmol m^{-2} d^{-1} determined from the pore water concentrations near the sediment-water interface (PW) and the benthic lander incubations (Lander).

Site	NH_4^+ [mmol m^{-2} d^{-1}] (PW)	NH_4^+ [mmol m^{-2} d^{-1}] (Lander)	HPO_4^{2-} [mmol m^{-2} d^{-1}] (PW)	HPO_4^{2-} [mmol m^{-2} d^{-1}] (Lander)	Fe [mmol m^{-2} d^{-1}] (PW)	Fe [mmol m^{-2} d^{-1}] (Lander)	Mn [mmol m^{-2} d^{-1}] (PW)	Mn [mmol m^{-2} d^{-1}] (Lander)
JML	2.94	N/A	0.18	N/A	-0.02	N/A	0.09	N/A
GOF5	1.16	0.93	0.20	-0.01	0.32	0.04	4.24	6.56
LL3A	2.34	1.63	0.06	1.04	0.05	1.07	4.25	3.67

1.10 Sedimentation Rates

Sedimentation rates were estimated by fitting a reactive transport model (Soetaert and Herman, 2008) to the ^{210}Pb depth profiles (Fig. S7) taking the depth dependent changes in porosity into account (Fig. S8).

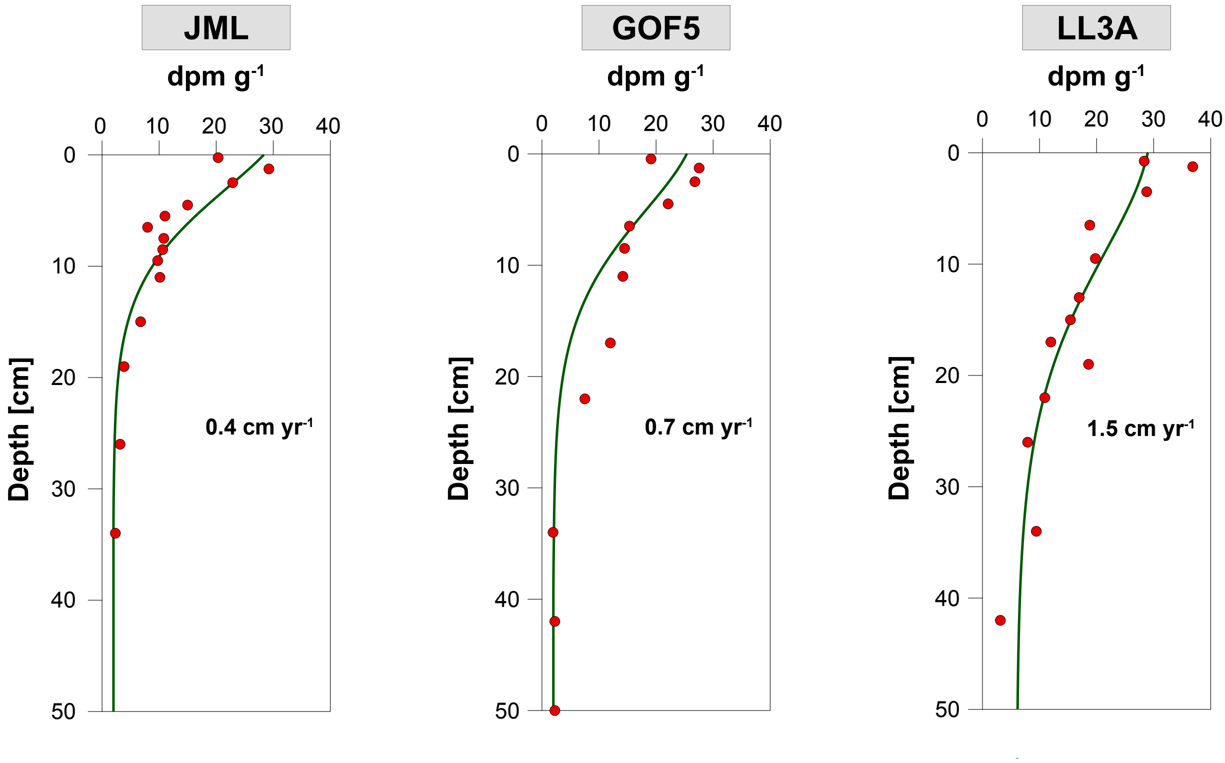

Figure S7. Sedimentation rates for sites JML, GOF5 and LL3A in cm yr^{-1}.

1.11 Porosity

The porosity (Fig. S8) was calculated from the water loss upon freeze-drying and sediment density following Burdige (2006).

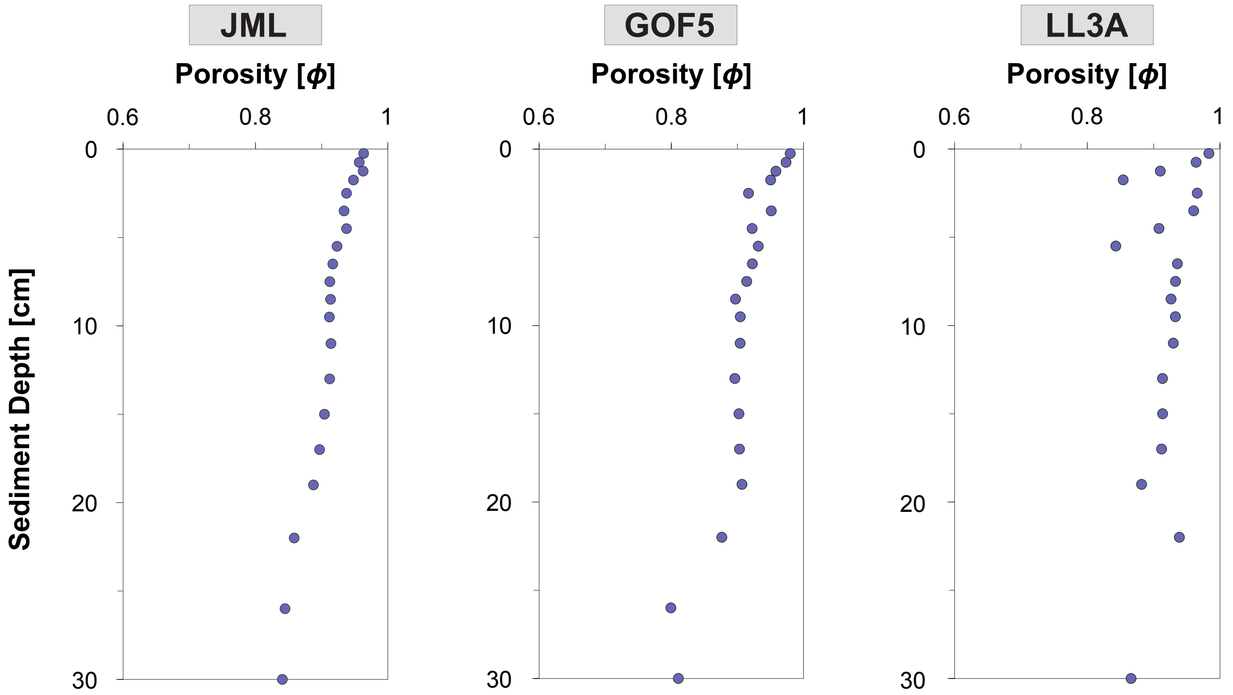

Figure S8. Porosity depth profiles for sites JML, GOF5 and LL3A in vol vol^{-1}.

1.12 Organic Carbon versus Organic Nitrogen and Sulfur

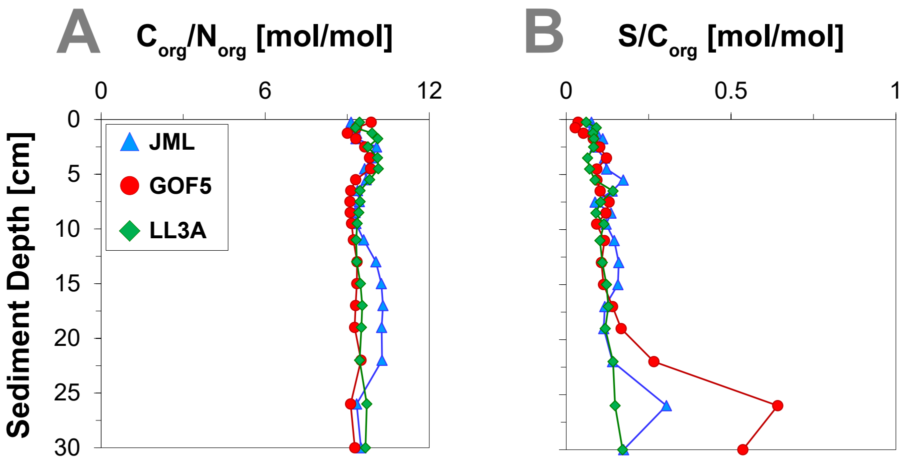

Figure S9. Sediment proxies: **(A)** C_{org}/N_{org} ratio in mol mol^{-1} **(B)** S/C_{org} ratio in mol mol^{-1} for sites JML, GOF5 and LL3A.

1.13 Sediment Mn proxy

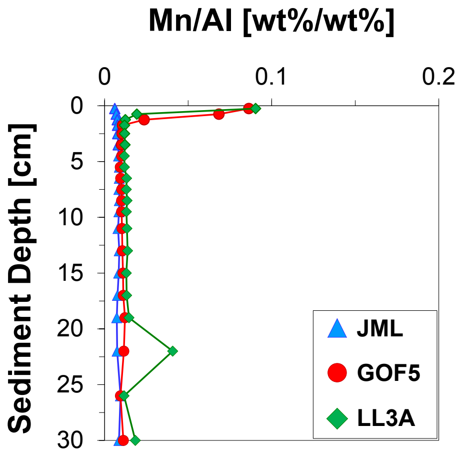

Figure S10. Sediment Mn proxies for sites JML, GOF5 and LL3A: Mn/Al ratio in wt% $wt\%^{-1}$.

1.14 Sediment Fe proxies

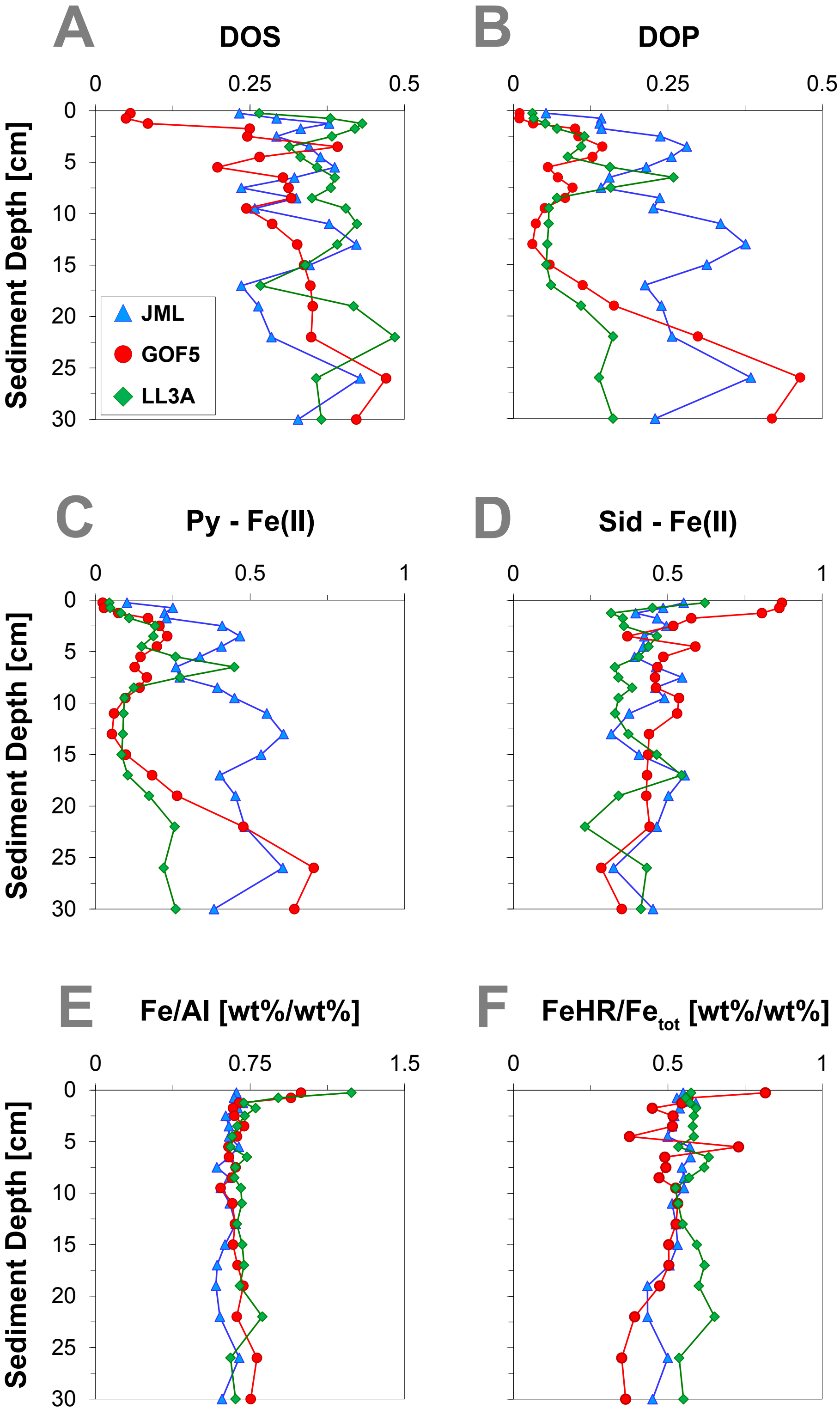

Figure S11. Sediment Fe proxies for sites JML, GOF5 and LL3A: **(A)** degree of sulfidization (DOS), **(B)** degree of pyritization, **(C)** Py - Fe(II), **(D)** Sid - Fe(II), **(E)** Fe/Al ratio (wt%/wt%) and **(F)** highly reactive Fe/total Fe (FeHR/Fe$_{tot}$) ratio (wt%/wt%).

1.15 Elemental Ratios in the Surface Sediment of Site GOF5

Table S3. Elemental ratios of Fe, Mn, P, Ca and S in the surface sediment of site GOF5.

Elements	Layer	Correlation [R^2]	Ratio [counts/counts]
Fe : P	Zone A	0.45	290 : 1
Fe : P	Zone B	0.49	75 : 1
Mn : P	Zone A	0.42	315 : 1
Mn : P	Zone B	0.61	5 : 1
Ca : P	Zone A	0.51	13 : 1
Ca : P	Zone B	0.78	3 : 2
Fe : Ca	Zone A	0.89	21 : 2
Fe : Ca	Zone B	0.67	29 : 1
Mn : Ca	Zone A	0.63	25 : 1
Mn : Ca	Zone B	0.68	7 : 2
S : Fe	Zone C	0.02	N/A
S : Fe	Zone D	0.55	3 : 8

N/A = not available

1.16 Fe XANES Spectra of Resin-embedded Sediment from Site GOF5

In contrast to Mn, the ratio between the reactive and unreactive fraction of Fe (i.e. Fe oxide and Fe sulfide versus clay Fe) was generally low in the sediments, even in enriched layers. As the size of the Fe enrichments was also smaller than the depth of the volume probed by XAS, the obtained Fe spectra also entail a contribution from non-reactive, silicate-bound Fe. In order to investigate the nature of Fe enrichments an attempt was made to isolate the contribution from non-reactive Fe. Based on the relative count intensities derived from synchrotron-based mapping of Fe in the surface sediment at site GOF5, spots containing very little Fe were selected, which are assumed to represent 'background Fe' (Fig. 9B,C). Subsequently, the XANES spectra of background Fe were averaged and compared with the XANES spectra of various Fe mineral reference materials (Fig. S12). Our findings indicate that background Fe did not resemble the XANES spectra of Fe oxides, Fe carbonates, Fe phosphates and Fe sulfides. However, the XANES and EXAFS spectrum of background Fe could be best reproduced by combining the XANES spectra of biotite (an Fe phyllosilicate mineral) and illite (Fe associated with clay) which supports the interpretation that the spectrum reflects silicate-bound Fe (Fig. S12). The isolated XANES spectrum from 'background Fe' was then used in combination with spectra of reactive Fe phases in the LCF to reproduce the XANES and EXAFS spectra collected at the various spots. For the XANES spectra the energy range between about 7110 to 7190 eV was used and for the k^2 weighted EXAFS spectra the k-range between 2 and 8 $Å^{-1}$ was used in the LCF. Within the set of spectra of reference materials, the combination with the spectra of 6L-ferrihydrite gave the best and consistent results when applied to the XANES and EXAFS spectra.

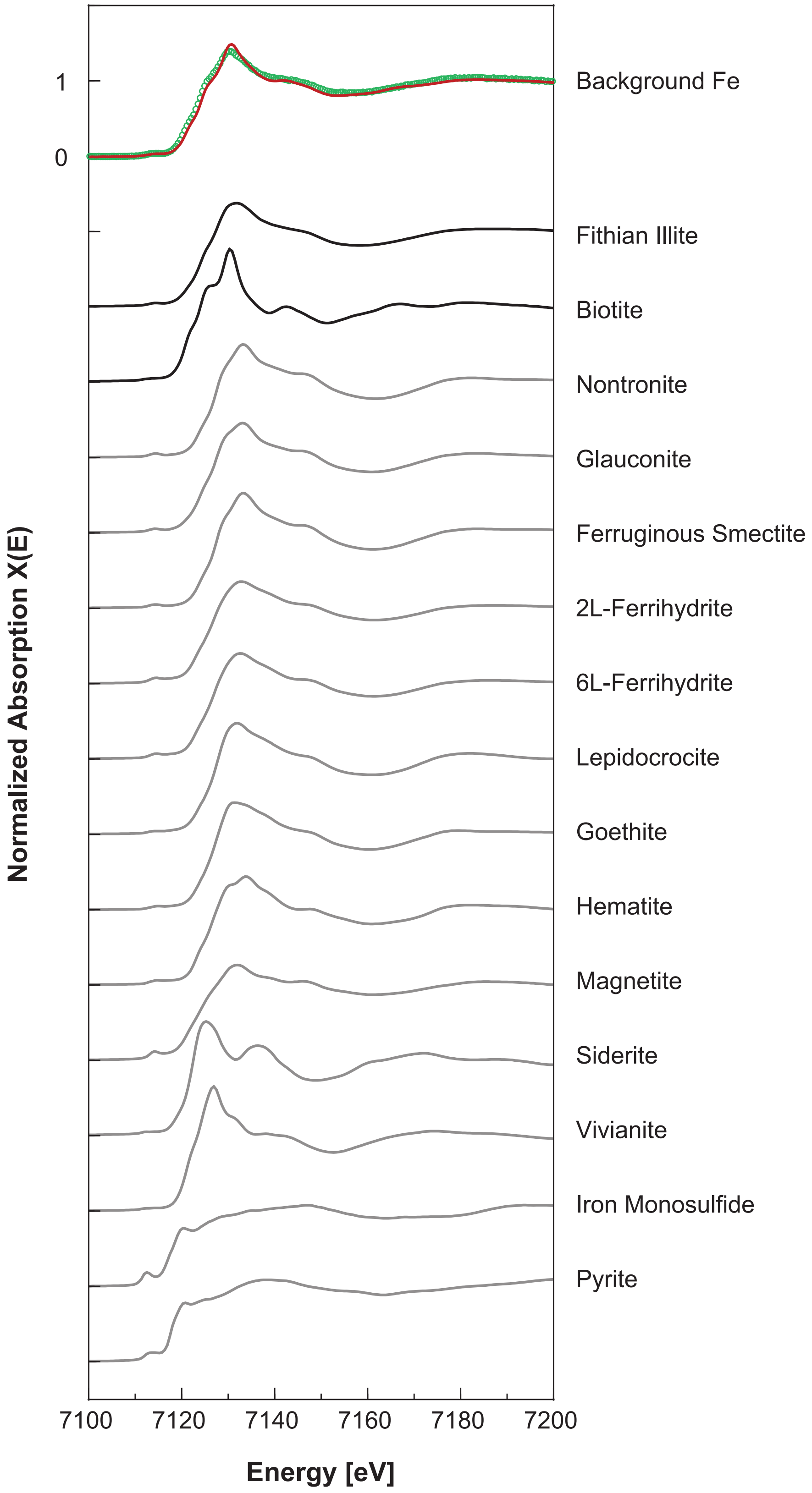

Figure S12. Normalized Fe XANES spectra of the background Fe and reference materials. The green circles represent the measured spectrum and the red line is the result from linear combination fitting using Fithian illite and biotite (black). The other spectra (grey) are from reference materials taken into account for the linear combination fitting.

1.17 Mn XANES Spectra of Resin-embedded Sediment from Site GOF5

In general, the approach for analyzing the Mn XANES spectra from sediments was similar to that used for suspended matter. In total 32 Mn XANES spectra collected from resin-embedded sediments of site GOF5 were used for the component analysis. The indicator function had a minimum at four components suggesting that the optimum number of endmember spectra for the reproduction of the sample spectra is four. Subsequently, the data set was extended by adding XANES spectra from reference materials to explore the effect on the indicator function and varimax rotation. All attempts to find a set of four spectra of reference materials without the need to increase the number of eigenvectors for maintaining the quality of reproductions did not succeed. This suggests that endmember spectra, which could be assigned to the components, might contain signals from more than one Mn phase. Another possibility is that one or more Mn phases that were present in the sediment are not included in the set of reference materials.

Based on these results, an approach was taken to include more than four spectra from reference materials in the LCF, in particular several spectra of solids containing Mn(II) and Mn(III). After testing several combinations, a good reproduction of all XANES spectra with a R-factor of 0.001 was achieved by using the spectra of the six reference materials: birnessite, manganite, bixbyite, hausmannite, Mn(II) phosphate, and rhodochrosite). However, due to the limitation of unambiguous identification of Mn phases based on Mn XANES spectra only, the results of the LCF were solely used to constrain the fractions of Mn(II) containing phosphates and carbonates, from Mn-containing oxides.

1.18 Fe, Mn and metal bound P pools at Site GOF5

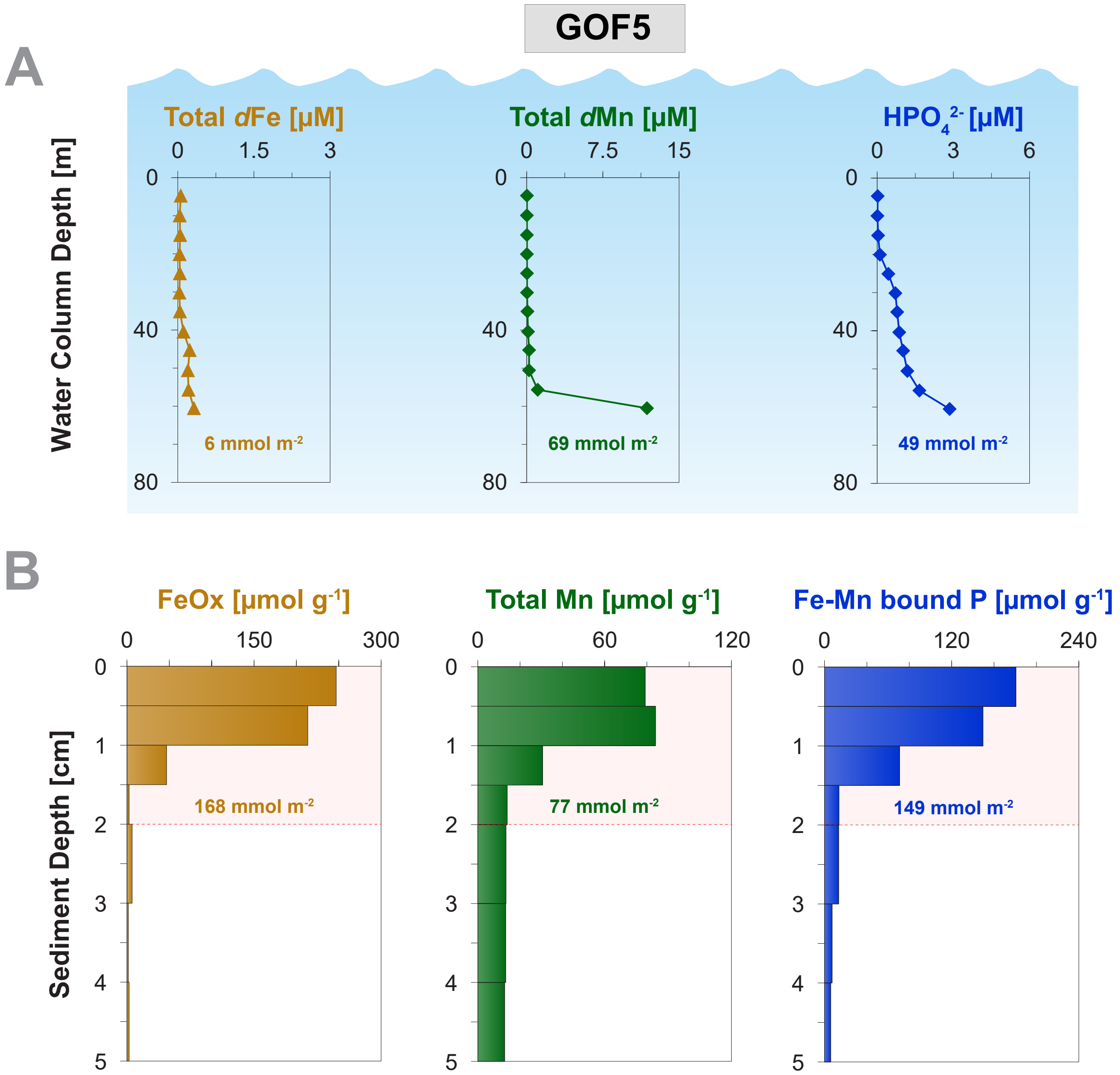

Figure S13. Fe, Mn, and P in the water column and seafloor at site GOF5 (**A**) Water column depth profiles of total dissolvable Fe, total dissolvable Mn and dissolved HPO_4^{2-}. The values reported in mmol m^{-2} indicate the total amount available in the water column by integrating the concentrations with water depth. (**B**) Solid-phase surface layer depth profiles of Fe oxides (FeOx), total Mn and Fe-Mn bound P. The values reported in mmol m^{-2} represent the total available pool of those species within the top 2 cm of the surface sediment.

1.19 Fe, Mn and metal bound P pools at Site LL3A

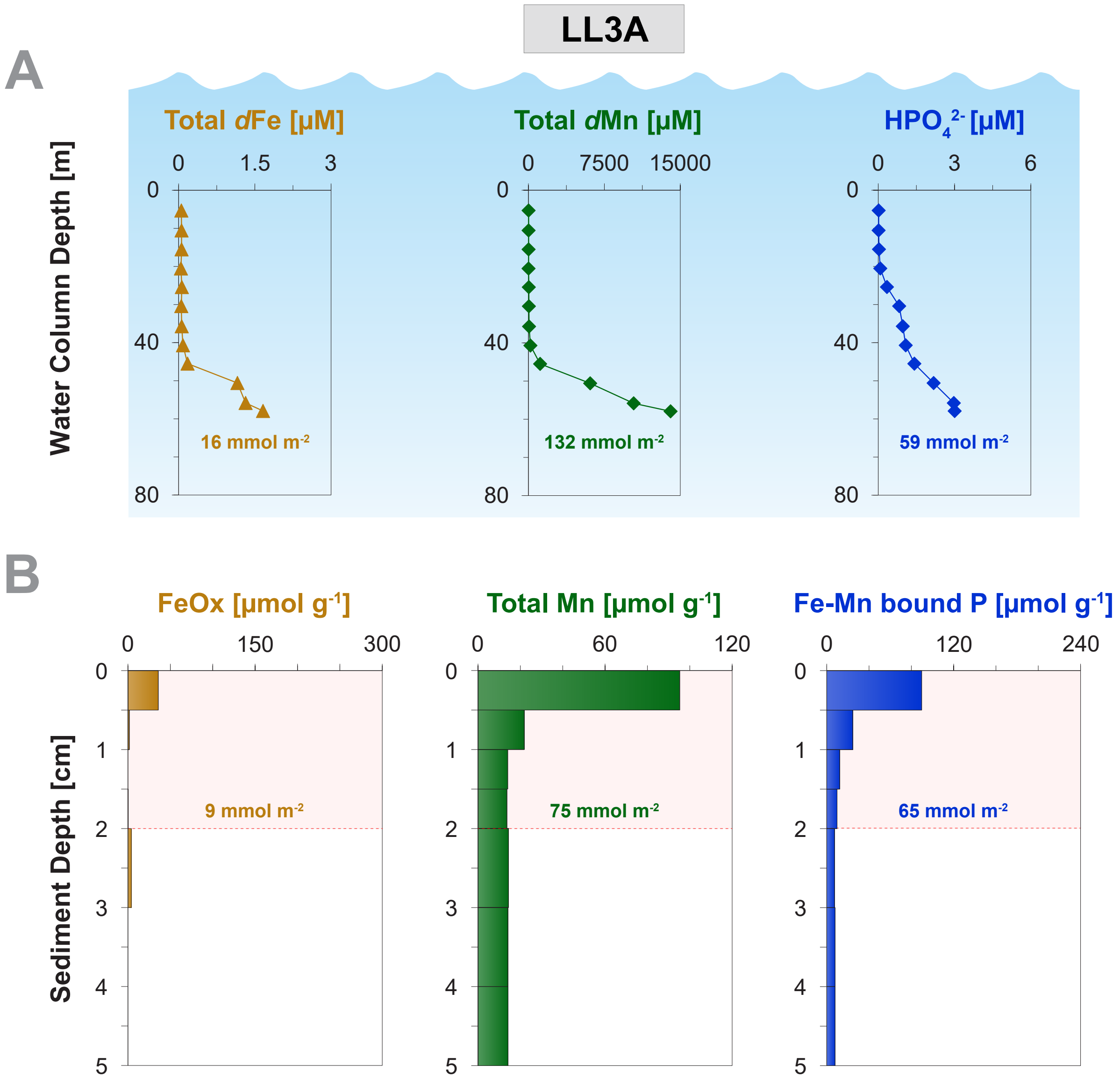

Figure S14. Fe, Mn, and P in the water column and seafloor at site LL3A (**A**) Water column depth profiles of total dissolvable Fe, total dissolvable Mn and dissolved HPO_4^{2-}. The values reported in mmol m^{-2} indicate the total amount available in the water column by integrating the concentrations with water depth. (**B**) Solid-phase surface layer depth profiles of Fe oxides (FeOx), total Mn and Fe-Mn bound P. The values reported in mmol m^{-2} represent the total available pool of those species within the top 2 cm of the surface sediment.

1.20 Phosphorus Burial in the Gulf of Finland Sediment

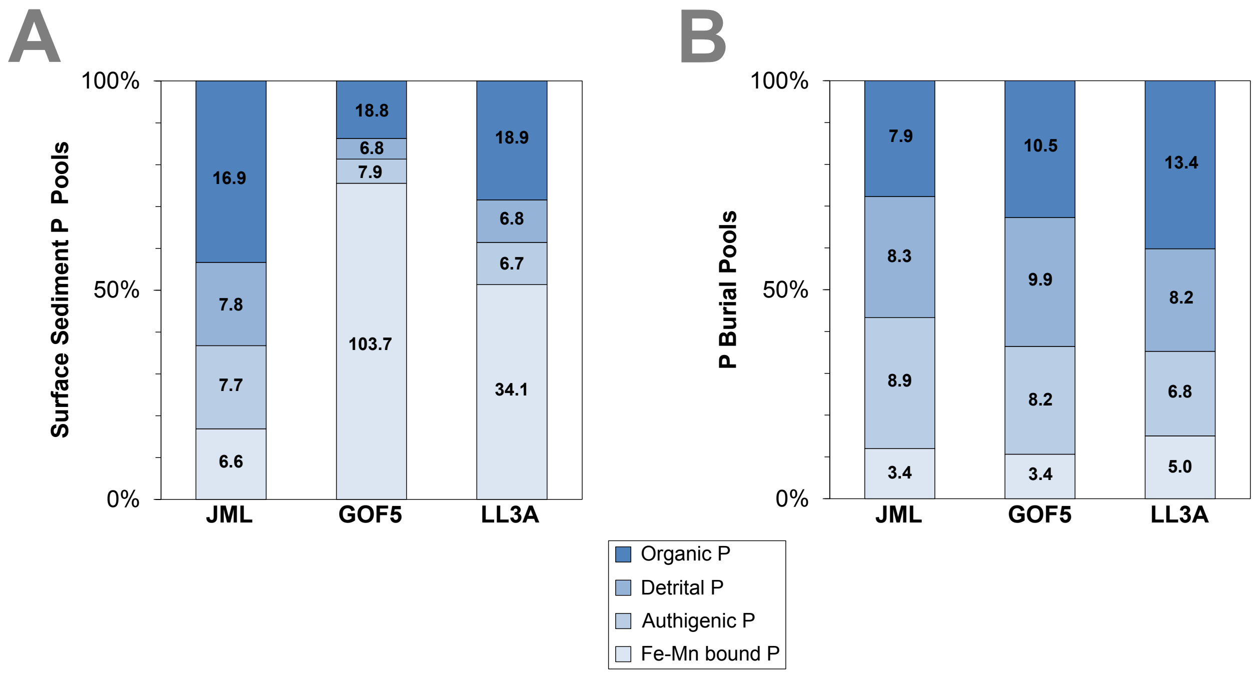

Figure S15. Phosphorus burial in the Gulf of Finland; (**A**) Sedimentary P pools in the upper 2 cm of the surface sediment. (**B**) Phosphorus burial pools in the deeper sediment (20-30 cm). The numbers are given in μmol g^{-1}.

1.21 Characteristic Sediment Features at Site GOF5

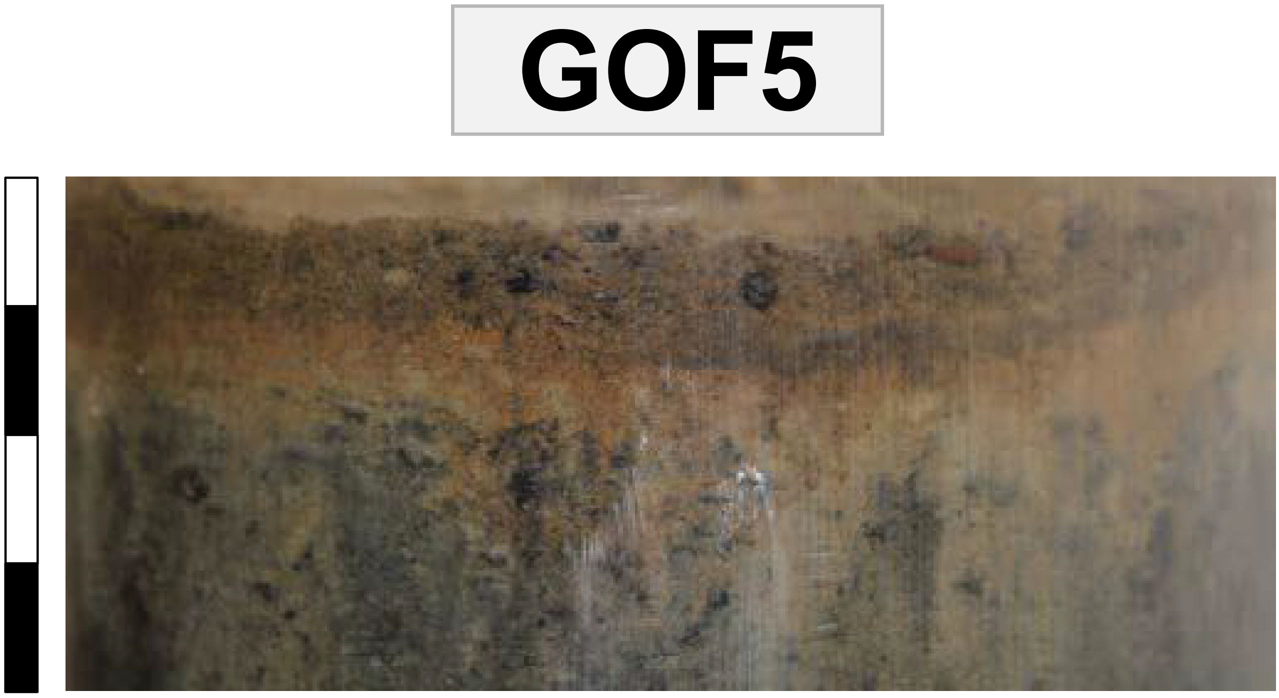

Figure S16. Photograph of the surface sediment at site GOF5, which is characterized by a distinct color zonation. The scale bar denotes a total distance of 2 cm, with 0.5 cm intervals.

1.22 Desktop µXRF Elemental Maps of Fe, Mn, P and S for Site JML

In contrast to sites GOF5 and LL3A, the surface sediment at site JML was not characterized by an enrichment of total Fe and Mn near the sediment-water interface (Fig. S17). However, total Fe, Mn, P and S do exibit vertical laminations, which is likely a consequence of the contrasting bottom water redox conditions over time.

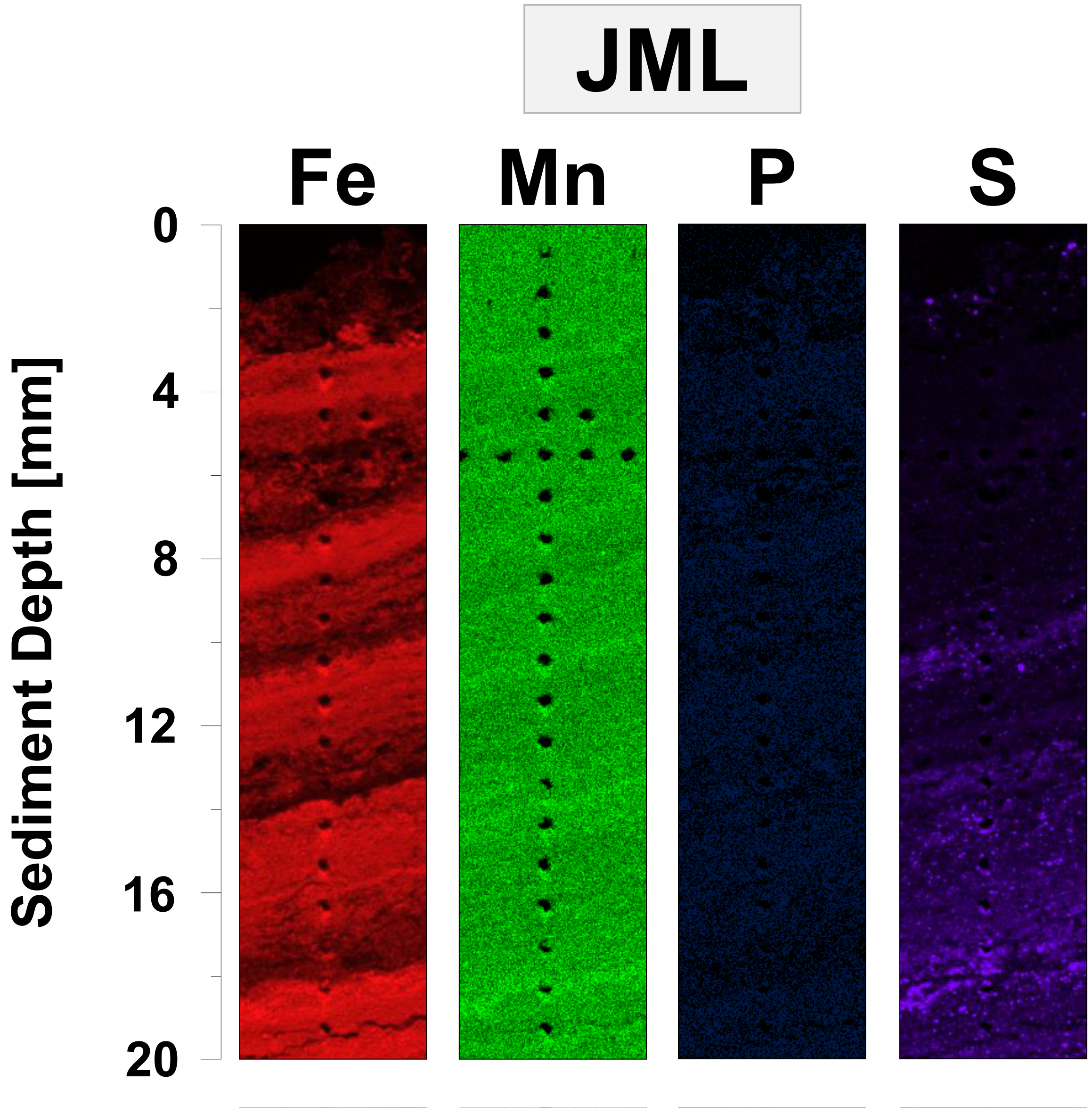

Figure S17. Desktop µXRF elemental maps of Fe, Mn, P and S for site JML in June 2016.

1.23 Distribution of Fe, Mn and P in Surface Layer of Site GOF 5

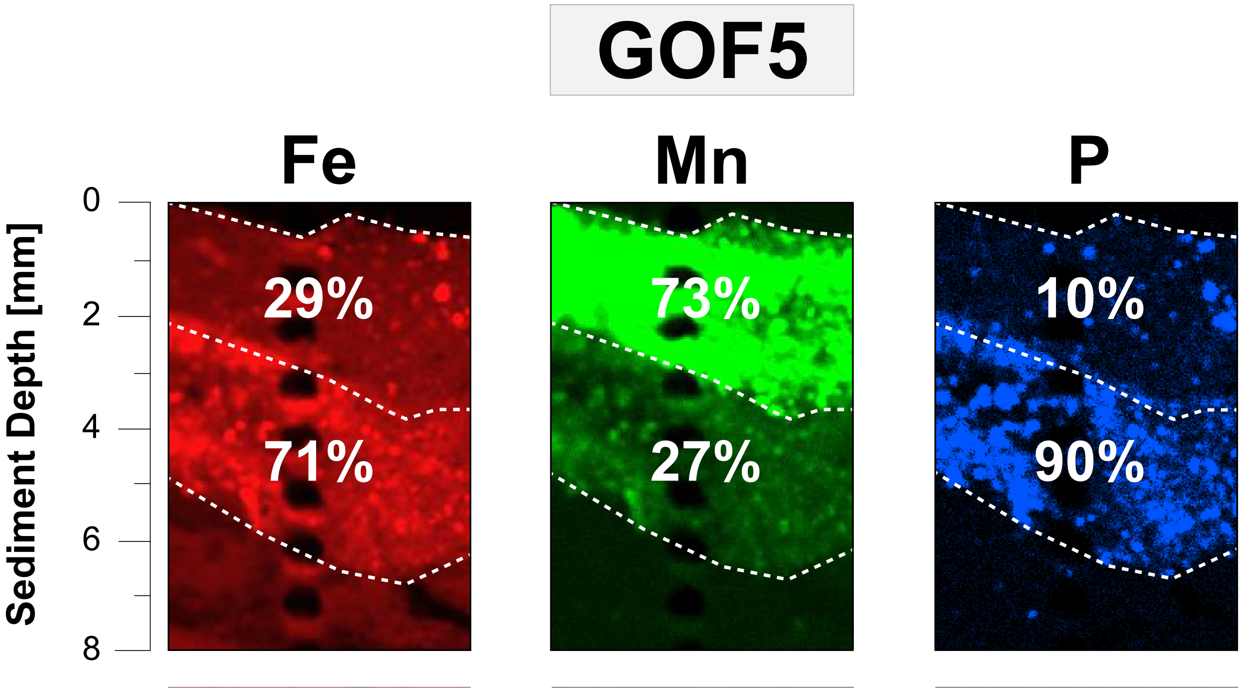

Figure S18. Desktop µXRF elemental maps, indicating the relative distribution of Fe, Mn and P in the top surface layer (0-2 mm) and the subsurface layer (2-5 mm). This distribution was determined from the relative count intensity of pixels.

1.24 Bottom Water O_2 versus HPO_4^{2-}

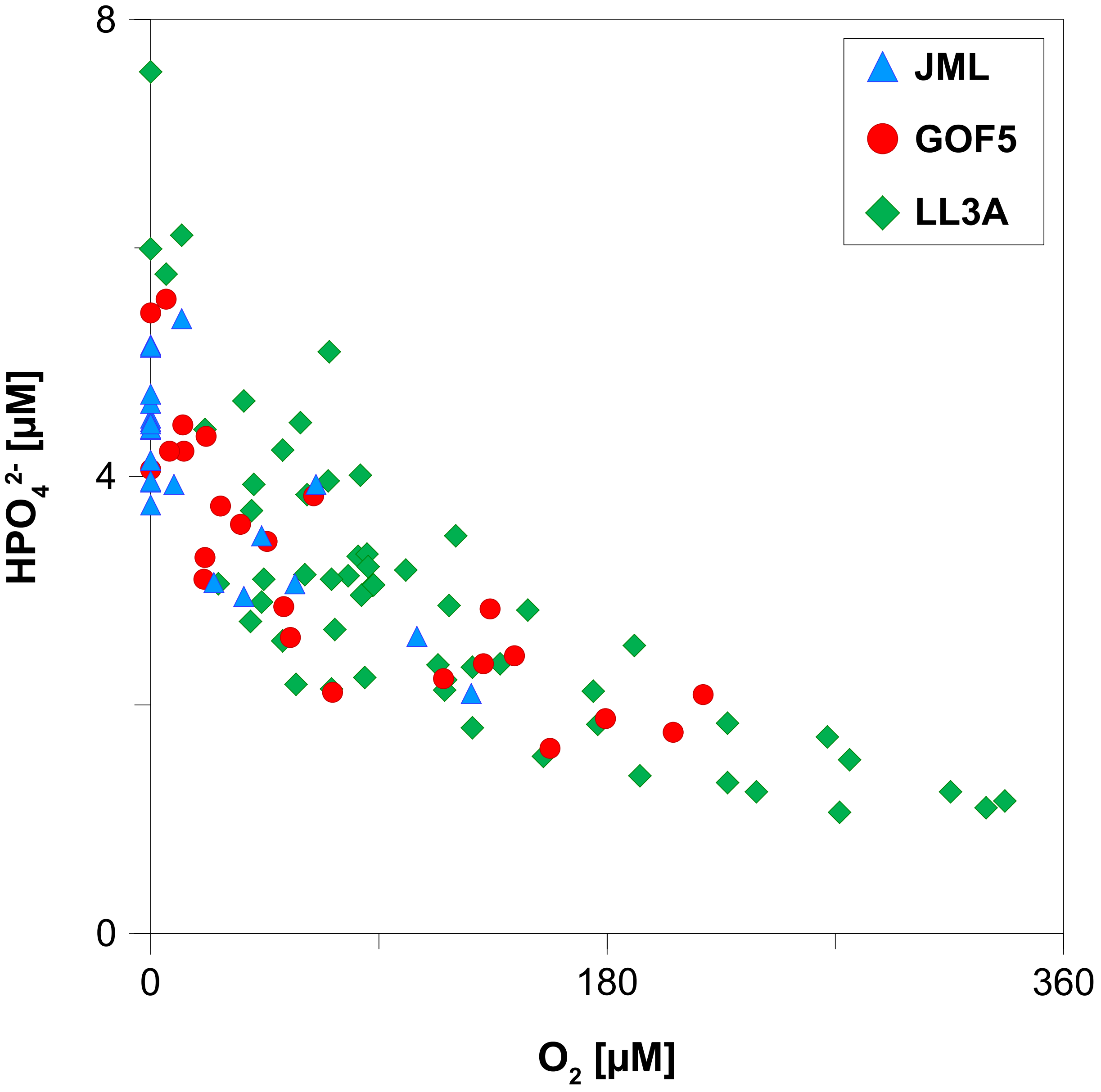

Figure S19. Relationship between bottom water O_2 and HPO_4^{2-} concentrations. This relationship was based on monthly time-series data (2002-2017) of dissolved bottom water O_2 and HPO_4^{2-} concentrations. These data were derived from the Swedish Ocean Archive (SHARK) database at the Swedish Meteorological and Hydrological Institute (SMHI; http://sharkweb.smhi.se).

1.25 Ammonium/Phosphate Ratios

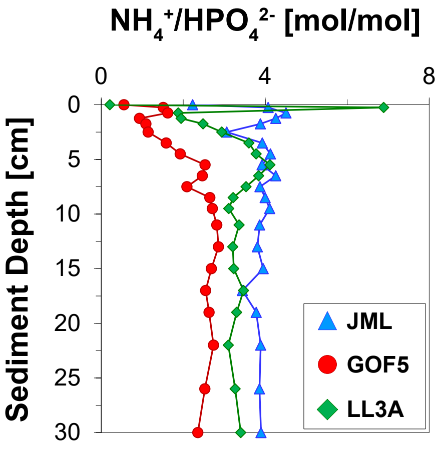

Figure S20. Pore water depth profiles of the NH_4^+/HPO_4^{2-} ratio for sites JML, GOF5 and LL3A.

1.26 Potential Change in Bottom Water HPO_4^{2-}

Table S4. Calculation of the potential change in bottom water P based on the Fe-bound P content of the surface sediment at site GOF5. The increase in HPO_4^{2-} of 7.4 µM was calculated by assuming release of all metal bound P from the upper 2 cm of the sediment at GOF5, to the lower 20 m of the water column, while accounting for the measured porosity in each sediment layer, assuming a sediment density of 2.65 g cm^{-3}. The total potential P release amounted to 150,000 µmol m^{-2}. When accounting for a volume of 20 x 1000 liters, this leads to a phosphate concentration of 7.4 µM. The actual change in the water column concentration (~4 µM) was calculated from the minimum and maximum P concentration for GOF5 as given in Fig. S19.

Sediment Depth Interval [cm]	Thickness of Depth Interval [cm]	Sediment Density [g/cm³]	Porosity [Φ]	Sediment Fraction [Φ]	Layer Volume per m² [cm³]	Layer Weight [g]	P Concentration [µmol/g]	Amount per m² [µmol]
0-0.5	0.5	2.65	0.98	0.02	5000	266	181	48063
0.5-1	0.5	2.65	0.97	0.03	5000	350	150	52358
1-1.5	0.5	2.65	0.96	0.04	5000	553	71	39152
1.5-2	0.5	2.65	0.95	0.05	5000	659	14	9030

References

1. Berner, R. A. 1980. Early diagenesis: a theoretical approach. Princeton University Press.
2. Boudreau, B. P. 1997. Diagenetic models and their implementation. Springer Berlin.
3. Burdige, D. J. 1993. The biogeochemistry of manganese and iron reduction in marine sediments. Earth-Science Reviews **35**: 249-284.
4. Rossberg, A., T. Reich, and G. Bernhard. 2003. Complexation of uranium (VI) with protocatechuic acid—application of iterative transformation factor analysis to EXAFS spectroscopy. Analytical and Bioanalytical Chemistry **376**: 631-638.
5. Soetaert, K. and Herman, P.M.J. A practical guide to ecological modelling: using R as a simulation platform. Springer Science & Business Media, 2008
6. Soetaert, K., T. Petzoldt, and F. Meysman. 2010. Marelac: Tools for aquatic sciences. R package version.
